# Supplementary material for: Rule-based omics mining reveals antimicrobial macrocyclic peptides against drug-resistant clinical isolates
Source: Nat Commun. 2024 Jun 8;15:4901. doi: 10.1038/s41467-024-49215-y (PMC11162475; doi:10.1038/s41467-024-49215-y)
Supplement: Supplementary file 1 — Supplementary Information [file 41467_2024_49215_MOESM1_ESM.pdf]

Rule-based omics mining reveals antimicrobial macrocyclic peptides against drug-resistant clinical isolates

Zhuo Cheng[1,2], Bei-Bei He[1], Kangfan Lei[3], Ying Gao[1], Yuqi Shi[1], Zheng Zhong[1], Hongyan Liu[1], Runze Liu[1], Haili Zhang[2], Song Wu[3], Wenxuan Zhang[3*], Xiaoyu Tang[2*] and Yong-Xin Li[1*]

[1]Department of Chemistry and The Swire Institute of Marine Science, The University of Hong Kong, Pokfulam Road, Hong Kong, China

[2]Institute of Chemical Biology, Shenzhen Bay Laboratory, Shenzhen, 515832, China

[3]State Key Laboratory of Bioactive Substance and Function of Natural Medicines, Institute of Materia Medica, Chinese Academy of Medical Sciences and Peking Union Medical College, Beijing 100050, China

[*]To whom correspondence may be addressed.

Email: wxzhang@imm.ac.cn; xtang@szbl.ac.cn; yxpli@hku.hk,

Table of content

Experiment materials and method 1

Strain and general biochemical reagents..1

Construction of single gene knockout strain *S. albus*/pCZMAT_*ΔR1*...........1

Construction of chimeric precursor mutant strains 2

Metabolites analysis using UPLC-HRMS 2

Isolation of Avi(Me)Cys-containing RiPPs 3

NMR analysis...4

Acid hydrolysis and advanced Marfey's analysis method 4

Bioactivity assay of the crude extract 5

Protease stability of massatide A 5

Thermal stability of massatide A 5

Mammalian cytotoxicity 5

Growth kinetics of *S. aureus*..6

Time-dependent killing assay 6

Resistance studies 6

Supplementary note 8

Mass calculation and mapping...8

Possible monoisotopic mass of both intact and fragmented Avi(Me)Cys compounds 8

Mass calculation and mapping workflow (using *mat* BGC as an example)..9

Supplementary tables 10

Table S1. Primers used in this study. 10

Table S2. Plasmids and strains in this study. 11

Table S3. Predicted functions for proteins in the BGCs in this study..........12

Table S4. ^{1}H and ^{13}C NMR data of massatide (**1**) in DMSO-*d6*..................13

Table S5. ^{1}H and ^{13}C NMR data of sistertide A1 (**2**) in DMSO-*d6*. 14

Table S6. ^{1}H NMR data of kebanetide A1 (**4**) in DMSO-*d6*. 15

Table S7. ^{1}H NMR data of kebanetide A2 (**5**) in DMSO-*d6*. 16

Table S8. Advanced Marfey's analysis for massatide (**1**). 17

Table S9. Advanced Marfey's analysis for sistertide A1 (**2**)........................18

Table S10. Advanced Marfey's analysis for kebanetide A1 (**4**)...................19

Table S11. Advanced Marfey's analysis for kebanetide A2 (**5**)...................20

Supplementary figures..21

Figure S1. Biosynthetic gene clusters of known Avi(Me)Cys-containing RiPPs. 21
Figure S2. Expanded cluster in precursor SSN. 24
Figure S3. UPLC-HRMS analysis of DSM42122 wild-type strain and heterologous expression of *mat* BGC. 25
Figure S4. Tandem MS/MS analysis for massatide A (**1**). 26
Figure S5. ^{1}H NMR spectrum (600 MHz, DMSO-*d6*) of massatideA (**1**)...27
Figure S6. ^{13}C NMR spectrum (150 MHz, DMSO-*d6*) of massatide A (**1**). 28
Figure S7. ^{1}H-^{1}H COSY spectrum (600 MHz, DMSO-*d6*) of massatide A (**1**). 29
Figure S8. TOCSY spectrum (600 MHz, DMSO-*d6*) of massatide A (**1**). 30
Figure S9. HMBC spectrum (600 MHz, DMSO-*d6*) of massatide A (**1**). 31
Figure S10. HSQC spectrum (600 MHz, DMSO-*d6*) of massatide A (**1**). 32
Figure S11. NOESY spectrum (600 MHz, DMSO-*d6*) of massatide A (**1**). 33
Figure S12. NMR analysis of massatide A. 34
Figure S13. Tandem MS/MS analysis for massatide B (**1a**). 35
Figure S14. UPLC-HRMS analysis of DSM40819 wild-type strain and heterologous expression of *sis* BGC. 36
Figure S15. Tandem MS/MS analysis for sistertide A1 (**2**). 37
Figure S16. ^{1}H NMR spectrum (600 MHz, DMSO-*d6*) of sistertide A1 (**2**). 38
Figure S17. ^{13}C NMR spectrum (150 MHz, DMSO-*d6*) of sistertide A1 (**2**). 39
Figure S18. ^{1}H-^{1}H COSY spectrum (600 MHz, DMSO-*d6*) of sistertide A1 (**2**). 40
Figure S19. TOCSY spectrum (600 MHz, DMSO-*d6*) of sistertide A1 (**2**). 41
Figure S20. HMBC spectrum (600 MHz, DMSO-*d6*) of sistertide A1 (**2**). 42
Figure S21. HSQC spectrum (600 MHz, DMSO-*d6*) of sistertide A1 (**2**). 43
Figure S22. NOESY spectrum (600 MHz, DMSO-*d6*) of sistertide A1 (**2**). 44
Figure S23. Purified compound **2**. Key COSY, HMBC, and NOESY correlations of sistertide A1 (**2**). 45
Figure S24. Tandem MS/MS analysis for sistertide A2 (**3**). 46
Figure S25. UPLC-HRMS analysis of DSM 42048 wild type strain and heterologous expression of *keb* BGC. 47
Figure S26. Tandem MS/MS analysis for kebanetide A1 (**4**). 48

Figure S27. Tandem MS/MS analysis for kebanetide A2 (**5**). 49
Figure S28. ^{1}H NMR spectrum (600 MHz, DMSO-*d6*) of kebanetideA1 (**4**). 50
Figure S29. ^{1}H-^{1}H COSY spectrum (600 MHz, DMSO-*d6*) of kebanetide A1 (**4**). 51
Figure S30. Key COSY correlations of kebanetide A1 (**4**). 52
Figure S31. ^{1}H NMR spectrum (600 MHz, DMSO-*d6*) of kebanetide A2 (**5**). 53
Figure S32. ^{1}H-^{1}H COSY spectrum (600 MHz, DMSO-*d6*) of kebanetide A2 (**5**). 54
Figure S33. Key COSY correlations of kebanetide A2 (**5**). 55
Figure S34. Chimeric precursor with maturase from *sis* BGC (constructs sis-syn1 and sis-syn2 design). 56
Figure S35. MS analysis for keb-syn1. 57
Figure S36. Retention time and tandem MS/MS analysis for keb-syn2. 58
Figure S37. Proteolysis stability of massatide A (**1**). 59
Figure S38. Thermal stability of massatide A (**1**). 60
Figure S39. Cytotoxicity of massatide A (**1**) and massatide B (**1a**). 61
Figure S40. The structural features, activity and synthesis of massatide A compared with other lanthipeptides in preclinical development. 62
References 63

Experiment materials and methods

Strain and general biochemical reagents

All the wild-type actinobacteria strains used in this study were purchased from DSMZ (https://www.dsmz.de/). *Streptomyces albus* J1074 was used as a heterologous expression host. *Escherichia coli* DH5α was used as a host for molecular cloning. *Escherichia coli* ET12567/pUZ8002 was used for biparental conjugation. Other plasmids and strains constructed in this study are listed in Table S2.

DNA polymerase Phanta Master Max Mix (Vazyme) was used for all polymerase chain reactions (PCR). Endonucleases and NEBuilder® HiFi DNA assembly were purchased from New England Biolabs (NEB). T4 DNA ligase was purchased from Vazyme. Oligonucleotide primers and chimeric precursor genes were synthesized from RuiBiotech (Guangzhou). Marfey's reagents were purchased from the Tokyo Chemistry Industry (TCI), L-FDLA(CAS: 178065-29-7, purity >98%), D-FDLA (CAS: 178065-30-0, purity >98%). Vancomycin was purchased from Macklin (CAS: 1404-90-6, purity >95%). Nisin was purchased from Sigma (CAS: 1414-45-5, potency≥900IU/mg). Norfloxacin was purchased from Bidepharm (CAS: 70458-96-7, purity: 99.88%). All culture mediums were purchased from Sigma and Oxoid. NMR solvent dimethyl sulfoxide-*d6* was purchased from Cambridge Isotope Laboratories (CAS: 2206-27-1, purity: 99.5%). NMR data were collected on Bruker Avance 600 MHz instruments.

Construction of single gene knockout strain *S. albus/*pCZMAT_*ΔR1*

*S. albus/*pCZMAT_*ΔR1* were constructed by λ-red mediated PCR targeting technolog[1]. Primers *Δ*matR1-EryB_F and *Δ*matR1-EryB_R were used to amplify the erythromycin resistance gene cassette from pUC57-Ery_FRT. The resistance gene cassette was used to transform *E. coli* BW25113/pIJ790/pCZMAT by electroporation to replace the *matR1* gene. We then extracted the plasmid of the successful replacement construct. *PacI* enzyme digestion and self-ligation were used to remove the resistance gene *eryB*. The knockout plasmid was confirmed by PCR using primer *Δ*matR1-ver_F and

*Δ*matR1-ver_R. pCZMAT_*ΔR1* was introduced to *S. albus* J1074 by bi-parental conjugation, yielding *S. albus/*pCZMAT_*ΔR1*.

Construction of chimeric precursor mutant strains

An in-situ replacement strategy was used to generate chimeric precursor mutant strains. pCZKEB was first digested with restriction enzymes *Asi*SI and *Bsr*GI to obtain a 21,525 bp backbone. A 6,560 bp fragment and a 6,301 bp fragment which contains the precursors, were amplified from pCZKEB using primer keb_BsrGI_pUC57_F/keb_R2-1 and keb_F3-1/ keb_AsiSI_pUC57_R, and then cloned into pUC57 vector by HiFi assembly to obtain pUC57-keb_AsiSI_BsrGI. The synthesized chimeric precursor gene *syn1* replaces the *kebA1* using PCR amplification and HiFi assembly, so we obtained pUC57-keb-syn1_AsiSI_BsrGI vector, including chimeric precursor *syn1* in the *keb* BGC fragment. Then, pUC57-keb-syn1_AsiSI_BsrGI was digested with restriction enzymes *Asi*SI and *Bsr*GI. The 12,754 bp fragment was insert into pCZKEB backbone as mentioned before to obtain pCZKEB-syn1. The chimeric mutant plasmid pCZKEB-syn1 was transferred into *S. albus* via bi-parental conjugation to obtain the chimeric precursor strains keb-syn1. Other chimeric mutant vectors pCZKEB-syn2, pCZSIS-syn1, and pCZSIS-syn2 were constructed by using a similar procedure. The chimeric precursor gene *syn1* replaced precursor gene *A1*, while *syn2* replaced precursor gene *A2*. To construct pCZSIS-syn, *Bsr*GI and *Avr*II double-digested pCZSIS was used as the backbone. The primers used in construction are listed in Table S1.

Metabolites analysis using UPLC-HRMS

The methanol-dissolved sample was centrifuged at 15,000 ×g for 10 min. The supernatant was collected and injected into a reverse phase column (Waters ACQUITY UPLC BEH C18, 130Å, 1.7 µm column) on Thermo Scientific UltiMate 3000 UHPLC system coupled with Bruker impact Mass Spectrometer (QTOF). The column temperature was 40 °C. H_2O (0.1% formic acid, A) and ACN (0.1% formic acid, B) were used as mobile phase at a flow rate of 0.2 mL/min. The UPLC method was: 0-2 min, 95% A / 5% B; 17 min, 5% A / 95% B; 17-21 min, 5% A / 95% B; 21-22 min, 95% A / 5% B. QTOF-MS was operated in positive mode. The MS system used the sodium

formate standard for calibration. MS data was analyzed by Bruker DataAnalysis 4.3 (×64).

Isolation of Avi(Me)Cys-containing RiPPs

For massatide A (**1**) isolation, *S. albus*/pCZMAT was cultivated on 2 L optimized GYM agar plate for 7 days at 30 °C. The compound extraction was performed as mentioned before. To obtain the pure compound **1**, the crude extract was dissolved in about 10 mL methanol and purified by HPLC with reverse phase semi-preparative column Kinetx 5 µm XB-C18 100 Å, 250 * 10 mm under the following conditions: mobile phase ACN/H_2O (0.5‰ TFA), 0-3 min, 70% H_2O/30% ACN; 21 min, 40% H_2O/60% ACN; 21-25 min, 100% ACN; 25-30 min, 70% H_2O/30% ACN. The flow rate was 3 mL/min. Massatide showed a peak at 16 min. For compound **1** large scale isolation, the crude extract was loaded onto an ODS column and eluted by water/methanol gradient. Fractions eluting with 100% methanol containing massatide A were collected and then used for further purification.

To isolate massatide B (**1a**), *S. albus*/pCZMAT_*ΔR1* was cultivated. The remaining fermentation and isolation methods followed the same procedure as for massatide A.

For sistertide A1(**2**) isolation, *S. albus*/pCZSIS was cultivated on 8 L R5A agar plate. Purification by HPLC also used ACN and H_2O as mobile phase under the following conditions: 0-3 min, 70% H_2O/30% ACN; 28 min, 50% H_2O/50% ACN; 28-31 min, 100% ACN; 31-34 min, 70% H_2O/30% ACN. The remaining conditions were kept the same as previously mentioned. Sistertide A1 was eluted at 18 minutes.

S. albus/pCZKEB was cultivated on a 4 L R5A agar plate for kebanetide A1 (**4**) and A2 (**5**) isolation. The preparative column RyoungC18-10 nm 5 um, 20*250 mm was first used to collect the fraction containing **4** and **5** at an 8 mL/min flow rate. The HPLC method was 0-5 min, 80% H_2O/20% ACN; 35 min, 40% H_2O/60% ACN; 35-40 min, 100% ACN; 40-45 min, 80% H_2O/20% ACN. The fraction from 28 to 33 min was collected and then freeze-dried, and the dried powder was dissolved in methanol. Two compounds were finally purified using a semi-preparative column with the following

conditions: 0-3 min, 70% H_2O/30% ACN; 40 min, 50% H_2O/50% ACN; 40-44 min, 100% ACN; 44-46 min, 70% H_2O/30% ACN. The column and flow rate employed were the same as those used for the isolation of massatide. Notably, Kebanetide A1 and A2 were eluted at 30 minutes and 22.5 minutes, respectively. All the purified compounds were freeze-dried.

NMR analysis

^{1}H NMR, ^{13}C NMR, HSQC, HMBC, ^{1}H-^{1}H COSY, TOCSY, and NOESY spectra were acquired on a Bruker Avance 600 MHz spectrometer with Cryoprobe, using dimethyl sulfoxide--*d*6 as solvent. The dimethyl sulfoxide-d6 chemical shifts were used as the internal reference.

Acid hydrolysis and advanced Marfey's analysis method

0.1 mg pure peptide sample was hydrolyzed with 1 mL 6 M HCl at 110 °C for 18 h. The hydrolyzed sample was dried under N_2 gas stream and dissolved in 90 μL water. Next, the sample was equally divided into two tubes. For each of the tubes, 15 μL 1 M $NaHCO_3$ was added. Finally, 100 μL 1% (acetone) L-FDLA and D-FDLA were respectively added in the two tubes, followed by incubation at 40 °C for 1 h. After cooling to room temperature, the reaction was quenched by adding 8 μL 2 M HCl and diluted with 200 μL MeOH. The reaction of the D-Abu standard was treated identically to the other samples. After centrifugation, 5 μL reaction mixtures were injected into an ultra-performance liquid chromatography-low resolution mass spectrometer (UPLC-LRMS). The column temperature was 40 °C. H_2O (0.1% formic acid, A) and ACN (0.1% formic acid, B) were used as mobile phase at a flow rate of 0.2 mL/min. The UPLC method was: 0-2 min, 95% A / 5% B; 20 min, 20% A / 80% B; 21-25 min, 100% B; 26-35 min, 95% A / 5% B. MS was operated in positive mode.The molecular mass corresponding to FDLA-amino acid derivatives was extracted for analysis.

For an amino acid X, the derivatives will be X-L-FDLA and X-D-FDLA. We can infer X based on K. Fujii et al (1997)[2]:
(1) the m/z determine the amino acid type and

(2) the retention time, if X-L-FDLA < X-D-FDLA, then X is L-configured. Otherwise, X is D-configured. Exceptions are argine, lysin and histidine, which exhibit opposite retention time.

Bioactivity assay of the crude extract

For the *S. albus*/pCZMAT crude extract, we did not concentrate the sample and simply utilized fermentation agar to test the activity (Figure 3e (i) and (ii)). For *S. albus*/pCZSIS, the crude extract was concentrated tenfold (Figure 4a).

Protease stability of massatide A

We selected TEV protease, Endoproteinase AspN, Endoproteinase GluC, trypsin, and chymotrypsin to evaluate the stability of massatide A. The protease digestion assay was conducted as follows: (i) Stock solution of massatide A (3.2 mg/mL in DMSO) was diluted in H_2O with final concentration of 32 μg/mL, (ii) each protease was added with 100:1 (w:w) ratio and incubated under corresponding temperature, i.e., 37 °C for TEV protease, trypsin, and chymotrypsin and 30 °C for Endoproteinase AspN and Endoproteinase GluC. Digested samples from three time points, 4h, 8h and 12h, were analyzed by UPLC-LRMS.

Thermal stability of massatide A

A solution of Massatide A (3.2 mg/mL) was prepared in 1.5 mL Eppendorf tube. These tubes were then incubated at 50 °C and 70 °C in a dry bath for 72 hours. After incubation, 1 μL samples were taken and diluted with water for UPLC-LRMS analysis.

Mammalian cytotoxicity

HeLa and Hek-293T cells were cultured in DMEM containing 10% fetal bovine serum and 1% penicillin-streptomycin. The cell lines were maintained at 37 °C in a humidity- and CO_2-controllable incubator with 5% CO_2. All the cell experiments were performed in a clean atmosphere.

Cell viability was determined by the CCK-8 assay. HeLa and Hek-293T cells were seeded into 96-well plates (5.0×10^3 cells per well) and incubated at 37°C for 24 h. The compound in DMEM was added at final concentrations of 200, 40, 8, 1.6, 0.32, 0.064

μg/ml. At the end of the fixed incubation period (24 h), cells were washed with PBS to remove excess compound. We added 100 μl fresh medium and 10 μl CCK-8 reagent. After 2 h incubation at 37°C, absorbance at 450 nm was recorded using a microplate reader. All experiments were performed in triplicate (n=3 independent experiments).

Growth kinetics of *S. aureus*

Massatide A or B was prepared in a 96-well plate at the concentration of 8×MIC, 4×MIC, 2×MIC, 1×MIC and 0.5×MIC, respectively. The MHB medium with no compound was used as a control. Then, the overnight culture of *S. aureus* ATCC 25923 cells was added to the medium at 1:1,000. The plate was incubated with shaking in SpectraMax iD3 (MOLECULAR DEVICES) at 30 °C. OD_{600} was measured every 30 min for 36 hours. To analyze the lysis, 1mL of *S. aureus* ATCC 25923 culture (OD_{600} 1.0) was treated with massatide A or vancomycin (10×MIC) for 24h. An untreated sample was used as a negative control.

Time-dependent killing assay

An overnight culture of *S. aureus* ATCC 25923 cells was diluted at 1:10,000 in MHB medium and incubated at 37 °C, 200 rpm for 2 hours to reach the early exponential. Then, massatide A or vancomycin was added to the culture at the concentration of 10×MIC. Bacteria were still cultivated at 37 °C, 200 rpm. Culture with no antibiotics was used as a negative control. 10 μL aliquots were removed at intervals, centrifuged, and resuspended in 1 ml PBS. Serially diluted (10-fold) suspensions were plated on MHB agar and incubated at 37 °C overnight for counting of colony-forming units. Experiments were performed in triplicate.

Resistance studies

Sequential culturing of *S. aureus* ATCC 25923 was performed in the presence of a subinhibitory concentration of massatide A to observe whether the resistance developed. *S. aureus* was grown in MHB medium overnight at 37 °C. Cells were diluted 1,000 times and then added to a 96-well plate containing massatide A or vancomycin or norfloxacin at different concentrations (from 16×MIC to 0.25×MIC). At 24-hour intervals, cultures from the second highest concentration that allow growth were

transferred to fresh MHB medium that contains massatide A or vancomycin or norfloxacin. The serial passaging was repeated for 25 days, and the MIC was confirmed. The experiment was performed in triplicate.

Supplementary note

Mass calculation and mapping

To predict the product of the BGC candidate, we need to enumerate all the precursor fragments and modifications. The monoisotopic mass and charge state of all the possible peptide fragments with different modifications, including decarboxylation, varied number of dehydrations, hydrogenations, and methylations, were calculated by using python script cal_lanv_ms1 (https://github.com/yxllab-hku/ACyPs_code/tree/main/ms-calculation). In this step, we will obtain a large m/z table.

The HRMS data of wild-type strain fermentation was used to match with the calculated *m/z*. Peptide signals in QTOF-MS usually show multiple charges, so we only focus on these signals and search them in the table to see whether they could match these calculated data with ppm<10. In this process, the charge state and biosynthetic logic were also used to exclude unreasonable cases. For instance, if the modification contains decarboxylation, then the sequence must end with cysteine; if the charge state was 2 in the mass data, we could only select calculated data with z=2. After this process, usually, one or several signals with predicted sequence and modification will be left.

Fragment ions with varied charge states and modifications of each putative core peptides were calculated by python script cal_lanv_ms2 (https://github.com/yxllab-hku/ACyPs_code/tree/main/ms-calculation). In this step, two tables will be generated, one for b-ion and another for y-ion. The matching process of MS/MS data is similar to MS1, and also need to consider the rationality between fragments. These including (1) the *m/z* value and charge state, (2) decarboxylation should consistant with the presene of C-ternimal Cys residue in b-ions or y-ions and (3) the number of predicted dehydrations should less or equal to the total number of Ser/Thr.

Possible monoisotopic mass of both intact and fragmented Avi(Me)Cys compounds

The mass calculation table for BGC in this study is in the supplementary dataset.

Mass calculation and mapping workflow (using *mat* BGC as an example)

Precursor:
MDTHELIEGFDAYVEAEELNEDAMVDAPATTVPCTVASFATGYFSC

↓ cal_lanv_ms1.py

cal_ms1_data, 1081 rows * 531 columns

	A	B	C	D	E	F	G
1	seq	isomass	1	2	3	-1H2O_-CO2H2_+0H_+0CH2_+1	-1H2O_-CO2H2_+0H_+1CH2_+1
2	M	149.0510493	150.0583258	75.5328011	50.6909596	86.0422818	100.0579319
3	MD	264.0779924	265.0852689	133.0462727	89.0332739	201.0692248	215.0848749
4	MDT	365.1256709	366.1329474	183.5701119	122.7158334	302.1169034	316.1325534
5	MDTH	502.1845828	503.1918593	252.0995679	168.4021374	439.1758152	453.1914653
6	MDTHE	631.2271759	632.2344524	316.6208644	211.4163351	568.2184084	582.2340584
7	MDTHEL	744.3112399	745.3185164	373.1628964	249.1110231	681.3024724	695.3181224
8	MDTHELI	857.3953040	858.4025804	429.7049284	286.8057111	794.3865364	808.4021865
9	MDTHELIE	986.4378971	987.4451736	494.2262250	329.8199088	923.4291295	937.4447796
10	MDTHELIEG	1043.4593608	1044.4666373	522.7369569	348.8270634	980.4505933	994.4662433
11	MDTHELIEGF	1190.5277748	1191.5350512	596.2711639	397.8498681	1127.5190072	1141.5346573
12	MDTHELIEGFD	1305.5547178	1306.5619943	653.7846354	436.1921824	1242.5459503	1256.5616003
13	MDTHELIEGFDA	1376.5918316	1377.5991081	689.3031923	459.8712203	1313.5830641	1327.5987141
14	MDTHELIEGFDAY	1539.6551602	1540.6624367	770.8348566	514.2256632	1476.6463927	1490.6620427
15	MDTHELIEGFDAYV	1638.7235742	1639.7308506	820.3690635	547.2484679	1575.7148066	1589.7304567
16	MDTHELIEGFDAYVE	1767.7661673	1768.7734438	884.8903601	590.2626656	1704.7573997	1718.7730498

exp_ms1_data

570.3312	3+	7.4min
521.3364	2+	9.8min
528.3439	2+	10.2min
504.8857	2+	10.6min
533.8520	2+	10.9min
817.8949	2+	10.9min
807.9002	2+	11.4min

← mapping, e.g., obs. m/z = 817.8949 $[M+2H]^{2+}$ from experiments

ms1_mapped_output_data

1	mapped_cal_m/z	cal_mod.	ppm	seq
2	817.8872	-6H2O_-CO2H2_+6H_+1CH2_+2	9.4	MDTHELIEGFDAYVE
3	817.8961	-4H2O_-CO2H2_+2H_+3CH2_+2	1.5	DTHELIEGFDAYVEA
4	817.8961	-4H2O_-CO2H2_+2H_+2CH2_+2	1.5	THELIEGFDAYVEAE
5	817.8961	-5H2O_-CO2H2_+6H_+1CH2_+2	1.5	HELIEGFDAYVEAEE
6	817.8978	-7H2O_-CO2H2_+10H_+2CH2_+2	3.5	AYVEAEELNEDAMVDA
7	817.8978	-7H2O_-CO2H2_+12H_+0CH2_+2	3.5	YVEAEELNEDAMVDAP
8	817.8902	-3H2O_-CO2H2_+4H_+2CH2_+2	5.8	VEAEELNEDAMVDAPA
9	817.8902	-4H2O_-CO2H2_+6H_+3CH2_+2	5.8	EAEELNEDAMVDAPAT
10	817.8902	-3H2O_-CO2H2_+2H_+4CH2_+2	5.8	AEELNEDAMVDAPATT
11	817.8902	-3H2O_-CO2H2_+2H_+2CH2_+2	5.8	EELNEDAMVDAPATTV
12	817.8995	-3H2O_-CO2H2_+2H_+3CH2_+2	5.6	EDAMVDAPATTVPCTVA
13	817.8965	-6H2O_-CO2H2_+6H_+2CH2_+2	2.0	TTVPCTVASFATGYFSC

↓ cal_lanv_ms2.py

cal_b_ions_data, 17 rows * 994 columns

1	b_ion_seq	-1H2O_+1	-1H2O_-0CO2H2_+1
2	T	84.04439027	84.04439027
3	TT	185.0920688	185.0920688
4	TTV	284.1604827	284.1604827
5	TTVP	381.2132466	381.2132466
6	TTVPC	484.2224311	484.2224311
7	TTVPCT	585.2701096	585.2701096
8	TTVPCTV	684.3385236	684.3385236
9	TTVPCTVA	755.3756374	755.3756374
10	TTVPCTVAS	842.4076658	842.4076658
11	TTVPCTVASF	989.4760797	989.4760797
12	TTVPCTVASFA	1060.513194	1060.513194
13	TTVPCTVASFAT	1161.560872	1161.560872
14	TTVPCTVASFATG	1218.582336	1218.582336
15	TTVPCTVASFATGY	1381.645664	1381.645664
16	TTVPCTVASFATGYF	1528.714078	1528.714078
17	TTVPCTVASFATGYFS	1615.746107	1615.746107
18	TTVPCTVASFATGYFSC	1718.755291	1718.755291

cal_y_ions_data, 17 rows * 994 columns

1	y_ion_seq	-1H2O_+1	-1H2O_-0CO2H2_+1
2	TTVPCTVASFATGYFSC	1736.766405	1736.766405
3	TVPCTVASFATGYFSC	1635.718726	1635.718726
4	VPCTVASFATGYFSC	1534.671048	1534.671048
5	PCTVASFATGYFSC	1435.602634	1435.602634
6	CTVASFATGYFSC	1338.54987	1338.54987
7	TVASFATGYFSC	1235.540685	1235.540685
8	VASFATGYFSC	1134.493007	1134.493007
9	ASFATGYFSC	1035.424593	1035.424593
10	SFATGYFSC	964.3874789	964.3874789
11	FATGYFSC	877.3554505	877.3554505
12	ATGYFSC	730.2870365	730.2870365
13	TGYFSC	659.2499227	659.2499227
14	GYFSC	558.2022442	558.2022442
15	YFSC	501.1807805	501.1807805
16	FSC	338.1174519	338.1174519
17	SC	191.049038	191.049038
18	C	104.0170095	104.0170095

ms2_mapped_output_data ← exp_ms2_data

1	ions	modified_seq	modification	z	observed m/z	calculated m/z	ppm
2	b5	TTVPC	-2H2O_+2CH2	1	494.2417	494.2432	3.0
3	b6	TTVPCT	-3H2O_+2CH2_+2H	1	579.2946	579.2959	2.2
4	b7	TTVPCTV	-3H2O_+2CH2_+2H	1	678.3636	678.3643	1.0
5	b8	TTVPCTVA	-3H2O_+2CH2_+2H	1	749.4004	749.4015	1.5
6	b9	TTVPCTVAS	-4H2O_+2CH2_+4H	1	820.4363	820.4386	2.8
7	b10	TTVPCTVASF	-4H2O_+2CH2_+4H	1	967.5063	967.5070	0.7
8	b11	TTVPCTVASFA	-4H2O_+2CH2_+4H	1	1038.5423	1038.5441	1.7
9	y6	TVASFATGYFSC	-2H2O_-CO2H2_+2H	1	597.2483	597.2495	2.0
10	y8	ASFATGYFSC	-2H2O_-CO2H2_+2H	1	815.3562	815.3551	1.3
11	y9	SFATGYFSC	-3H2O_-CO2H2_+4H	1	886.3907	886.3922	1.7
12	y10	FATGYFSC	-3H2O_-CO2H2_+4H	1	957.4333	957.4293	4.2
13	y11	ATGYFSC	-3H2O_-CO2H2_+4H	1	1056.4962	1056.4977	1.4

↓

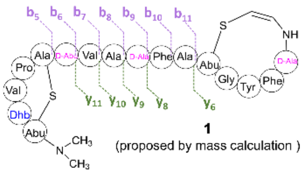

1
(proposed by mass calculation)

1. Precursor input with arguments

Modification types as arguments	mass change (Da)
Dehydration	-18.0105
Decarboxylation	-46.0055
Hydrogenation	+2.0156
Methylation	+14.0156
Cyclization	0

2. Calculated monoisotopic mass

All the fragments with different charge states and modifications.
For mat BGC precursor, the results contain 1081 rows * 531 columns

3. Collected HRMS data from extracts

All the experimental m/z candidates with monoisotopic mass and charge state.

- wild type extract
- medium extract as negative control

4. MS1 mapping by ppm and charge state

For m/z = 817.8949 $[M+2H]^{2+}$, there are 12 candidates, as shown left. Only keep calculated m/z candidates that have ppm < 10 and the same charge state with the observed m/z.

5. Further filtering and MS/MS calculation

Number of T and S in the sequences denotes the upper limit of dehydrations. E.g.,

- Decarboxylation requires one C-terminal Cys
- Dehydration requires Ser and Thr

After further filtration, the remaining sequences with predicated modifications were used for tandem mass calculation

6. MS/MS mapping

Mapping calculated MS/MS with calculated MS/MS will provide information regarding

- the modifications of each amino acids or small motifs
- structure illustration

7. Predicted core peptide with modifications

Based on the experimentally detected fragment ions, the structure of ACP could be proposed

Supplementary tables

Table S1. Primers used in this study.

Primer	Sequence
pJTU2554-F1	GATCCACTAGTTCGACCGATGCCCTTGAGAGCC
pJTU2554-R1	ATCCCTTAACGTGAGTTTTCGTTCCACT
pJTU2554-F2	AGTGGAACGAAAACTCACGTTAAGGGAT
pJTU2554-R2	CGATATCTCTAGAGTCGACCTGCAGCCCAAGC
mat_2554_F1-1	TTGGGCTGCAGGTCGACTCTAGAGATATCGGCGGCGCGTTCCCCGTTC
mat_2554_R1-1	GGCAGGGGTGGCGGAAGGACGAC
mat_F2-2	GCACCCCTTCGTCCGGGCTTGC
mat_R2-2	GTCCGGACGTCGTCATGCGGTGC
mat_2554_F3-1	CGCGGGAACAGACGGTGACGGAAC
mat_2554_R3-1	TCTCAAGGGCATCGGTCGAACTAGTGGATCACGGGTGCCGTTGCGGGTGC
⊿matR1-EryB_F	CGTCCTGAAGAGGTTCGGGACCCCGACCCCGGAGCGTGACGACGCTTAATTAAGA
⊿matR1-EryB_R	CGTGACCGTGGCGCGGCCGTGACCGGCGCGGGCCCGGCCGCCGGATTAATTAAAA
⊿matR1-ver_F	CGCCGGGTGGGTGGTGCTG
⊿matR1-ver_R	CGTACGGCGGGTGACGCACC
sis_2554_F1-1	TTGGGCTGCAGGTCGACTCTAGAGATATCGGTACCCGTCCGGTTGATGTGCGAGCG
sis_2554_R1-1	GGTTCCCCCAGGTGTGTTGCGGTGC
sis_F2-1	CGTCGGCACCGCAACACACCTGG
sis_R2-1	GACCTGCGGAGACCTCGACGCCAGG
sis_2554_F3-1	GACATCGGCGCTTGCGGCCCTG
sis_2554_R3-1	TCTCAAGGGCATCGGTCGAACTAGTGGATCGGTAGGTGGCAGGCGCAGGCAGG
keb_2554_F1-1	TTGGGCTGCAGGTCGACTCTAGAGATATCGGCACGTGTTCAGCACCGCTTCGTTCC
keb_2554_R1-1	GTGGAACGGACTCAAGTGTGCCTGAGGCTC
keb_F2-1	GTCTGTTGACCCGGCGGTGAGCCTCAG
keb_R2-1	CTCGATTTCCCTCTGCACGAAGATTTTCTCAGTGTTGC
keb_F3-1	GGAGTACGCCCGGCAACACTGAGAAAATCTTCGTG
keb_R3-1	CCATGTCCGGTTTCCCTCCCTGACGAGC
keb_2554_F4-1	GAGCTGGTCCGAGAGCTCGTCAGGGAGG
keb_2554_R4-1	TCTCAAGGGCATCGGTCGAACTAGTGGATCGACCGGGCAACAGCGCCCAGC
puc57_F	GGCGTAATCATGGTCATAGCTGTTTCCTG
puc57_R	ACTGGCCGTCGTTTTACAACGTC
keb_BsrGI_pUC57_F	CGACGTTGTAAAACGACGGCCAGTCCGATGAACTCGATGTACATCTCGAACGGCAG
keb_AsiSI_pUC57_R	AAACAGCTATGACCATGATTACGCCGCGATCGCCGCCAGCATCAGCG
sis_BsrGI_pUC57_F	CGACGTTGTAAAACGACGGCCAGTTGTACAACGAGCTCAGCTTCGCCGAGTTGGA
sis_AvrII_pUC57_R	AAACAGCTATGACCATGATTACGCCCCTAGGCCGAGAACTCGATCAACGACCGGAC
kebA1_change_F	CCCGCCCCTGACCCTGCCGAC
kebA1_change_R	GATTCTCTCCCTCTCCATTTTCGTTTCTGCTCTTTTCCAGAATCG
kebA2_change_F	TCGCAGCTCGTCGGCCGGC
kebA2_change_R	GGGTTCTCCCTCTCTTCATGCGCTCCCTACG
sisA1_change_F	ACCACCCGCAGCACCTTTTC
sisA1_change_R	GAATATCCCTCTCGTGGAATTCTGAGATGAATCC
sisA2_change_F	ACCTCCGTTCCGAGTGACCG
sisA2_change_R	GGCTTCACATCCTTCGGAAATGTCG
syn1_keb1_F	AACGAAAATGGAGAGGGAGAGAATCATGGACAAGTCCATGGCCATCATG
syn1_keb1_R	TGCGTCGGCAGGGTCAGGGGCGGGTCAGCAGGTCCACTCGTACGTCGC
syn2_keb2_F	GAGCGCATGAAGAGAGGGAGAACCCATGGAGAAGACCACCGCGATCATGGAG
syn2_keb2_R	CCACGGCCGGCCGACGAGCTGCGATCAGCAGCCGTCCTTGACGG
syn1_sis1_F	TCAGAATTCCACGAGAGGGATATTCATGGACAAGTCCATGGCCATCATG
syn1_sis1_R	GGTGGGAAAAGGTGCTGCGGGTGGTTCAGCAGGTCCACTCGTACGTCGC
syn2_sis2_F	CGACATTTCCGAAGGATGTGAAGCCATGGAGAAGACCACCGCGATCATGGAG
syn2_sis2_R	GGAGCCGGTCACTCGGAACGGAGGTTCAGCAGCCGTCCTTGACGG
syn3_sis1_R	GGTGGGAAAAGGTGCTGCGGGTGGTTCAGCAGCCGGCCTCGTAGG
syn4_sisi2_R	GGAGCCGGTCACTCGGAACGGAGGTTCAGCAGCCTTCCTTGTACGTCTTG

Table S2. Plasmids and strains in this study.

**Plasmids**	**Characteristic**	**Source**
pJTU2554	Integrative vector, attP$^{\Phi C31}$, *aac(3)IV*	[3]
pUC57	Basic cloning vector, *bla*	RuiBiotech
pUC57-Ery_FRT	pUC57 vector containing *eryB* gene	RuiBiotech
pCZMAT	pJTU2554 containing *mat* BGC	This study
pCZMAT_*ΔR1*	pCZMAT_*ΔR1*	This study
pCZSIS	pJTU2554 containing *sis* BGC	This study
pCZKEB	pJTU2554 containing *keb* BGC	This study
pUC57-keb_AsiSI_BsrGI	pUC57 containing *keb* BGC fragment	This study
pUC57-sis_AvrII_BsrGI	pUC57 containing *sis* BGC fragment	This study
pUC57-keb-syn1_AsiSI_BsrGI	*syn1* replace *kebA1* in pUC57-keb_AsiSI_BsrGI	This study
pUC57-keb-syn2_AsiSI_BsrGI	*syn2* replace *kebA2* in pUC57-keb_AsiSI_BsrGI	This study
pUC57-sis-syn1_AvrII_BsrGI	*syn1* replace *sisA1* in pUC57-sis_AvrII_BsrGI	This study
pUC57-sis-syn2_AvrII_BsrGI	*syn2* replace *sisA2* in pUC57-sis_AvrII_BsrGI	This study
pCZKEB-syn1	*syn1* replace *kebA1* in pCZKEB	This study
pCZKEB-syn2	*syn2* replace *kebA2* in pCZKEB	This study
pCZSIS-syn1	*syn1* replace *sisA1* in pCZSIS	This study
pCZSIS-syn2	*syn2* replace *sisA2* in pCZSIS	This study
**Strains**	**Characteristics**	**Source**
DSM42122	Wild type strain containing *mat* BGC	DSMZ
DSM40819	Wild type strain containing *sis* BGC	DSMZ
DSM42048	Wild type strain containing *keb* BGC	DSMZ
S. albus J1074	Heterologous expression host	[4]
S. albus/pJTU2554	*S. albus* with pJTU2554 empty vector in $attB_{\Phi C31}$	This study
S. albus/pCZMAT	*S. albus* with pCZMAT in $attB_{\Phi C31}$	This study
S. albus/pCZMAT_*ΔR1*	*S. albus* with pCZMAT_*ΔR1* in $attB_{\Phi C31}$	This study
S. albus/pCZSIS	*S. albus* with pCZSIS in $attB_{\Phi C31}$	This study
S. albus/pCZKEB	*S. albus* with pCZKEB in $attB_{\Phi C31}$	This study
keb-syn1	*S. albus* with pCZKEB-syn1 in $attB_{\Phi C31}$	This study
keb-syn2	*S. albus* with pCZKEB-syn2 in $attB_{\Phi C31}$	This study
sis-syn1	*S. albus* with pCZSIS-syn1 in $attB_{\Phi C31}$	This study
sis-syn2	*S. albus* with pCZSIS-syn2 in $attB_{\Phi C31}$	This study
M. luteus DSM1790	G+ indicator strain	DSMZ
B. subtilis 168	G+ indicator strain	Preserved in Li lab
S. aureus ATCC25923	G+ indicator strain	ATCC
E. faecalis OG1RF	G+ indicator strain	From Prasanna lab
E. faecium MCC2763	G+ indicator strain	From Prasanna lab
P. aeruginosa PAO1	G- indicator strain	Preserved in Li lab

Table S3. Predicted functions for proteins in the BGCs in this study.

orf	**Product size (aa)**	**Conserved domain**	**Proposed function**
matA	46	LxmA leader domain family RiPP	Precursor peptide
matD	211	Flavoprotein, PF02441	Cysteine decarboxylase
matX	298	Hypothetical protein	Involving cyclization
matK	377	Class V lanthionine synthetase subunit	Ser/Thr kinase
matY	330	Hypothetical protein	Putative lyase [5]
matM	341	O-methyltransferase domain	Methyltransferase
matR1	331	LLM class flavin-dependent oxidoreductase	Oxidoreductase
matR2	321	LLM class flavin-dependent oxidoreductase	Oxidoreductase
matP	841	Insulinase family protein	Peptidase
matT1	333	ABC transporter ATP-binding protein	Transporter
matT2	255	ABC transporter permease	Transporter
matT3	590	ABC transporter ATP-binding protein/ permease	Transporter
sisA1	68	LxmA leader domain family RiPP	Precursor peptide
sisA2	58	LxmA leader domain family RiPP	Precursor peptide
sisD	491	Flavoprotein, PF02441	Cysteine decarboxylase
sisK	387	Class V lanthionine synthetase subunit	Ser/Thr kinase
sisY	327	HopA1 effector protein family	Putative lyase [5]
sisM	335	O-methyltransferase domain	Methyltransferase
sisR	320	LLM class flavin-dependent oxidoreductase	Oxidoreductase
sisP	907	Insulinase family protein	Peptidase
sisT	603	ABC transporter ATP-binding protein/permease	Transporter
kebA1	64	LxmA leader domain family RiPP	Precursor peptide
kebA2	60	LxmA leader domain family RiPP	Precursor peptide
kebD	253	Flavoprotein, PF02441	Cysteine decarboxylase
kebX	284	Hypothetical protein	Involving cyclization
kebK	376	Class V lanthionine synthetase subunit	Ser/Thr kinase
kebY	340	HopA1 effector protein family	Putative lyase
kebM	337	O-methyltransferase domain	Methyltransferase
kebR	346	LLM class flavin-dependent oxidoreductase	Oxidoreductase
kebP	875	Insulinase family protein	Peptidase
kebT1	296	ATP-binding cassette domain-containing protein	Transporter
kebT2	390	ABC transporter permease	Transporter
kebT3	597	ABC transporter ATP-binding protein/permease	Transporter

*The assetion ID and sequence information are in the supplymentary data 8.

Table S4. ^{1}H and ^{13}C NMR data of massatide (**1**) in DMSO-*d6*.

	Position	δC	δH, mult(*J* in Hz)		Position	δC	δH, mult(*J* in Hz)
**NMe2-Thr-1**	1	167.3, C		**Phe-10**	1	170.4, C	
	2	71.0, CH	4.01, d (4.8)		2	53.4, CH	4.58
	3	40.1, CH	3.67,		3	37.7, CH_2	2.65/3.06
	4	15.6, CH_3	1.57, d (4.3)		4	137.9, C	
	$N(CH_3)_2$	41.4, CH_3	2.81, s		5/9	129.3, CH	7.22
	$N(CH_3)_2$	43.9, CH_3	2.76, s		6/7/8	127.9, CH	7.24
**Dhb-2**	1	163.3, C			NH		8.20, d (8.9)
	2	130.3, C		**Ala-11**	1	171.7, C	
	3	126.2, CH	6.32, q (6.5)		2	48.27, CH	4.33
	4	12.8, CH_3	1.71, d (6.9)		3	18.3, CH_3	1.26, d (7.1)
	NH		9.87, s		NH		8.23, d (7.2)
**Val-3**	1	171.0, C			1	170.5, C	
	2	57.6, CH	4.05		2	55.9, CH	4.34
	3	29.5, CH	2.04	**Thr12***	3	45.3, CH	3.23
	4	19.5, CH_3	0.96, d (6.4)		3’	19.6, CH_3	1.21
	4’	18.4, CH_3	0.84, m		NH		8.42, d (9.0)
	NH		8.06		4	105.2, CH	5.29, d (8.5)
**Pro-4**	1	170.4, C		**Cys17***	5	121.9, CH	6.66, q (8.4)
	2	63.1, CH_2	3.35/3.29		5-NH		8.68, d (9.5)
	3	30.9, CH_2	1.84,	**Gly-13**	1	167.3, C	
	4	21.9, CH_2	2.07/1.74		2	42.4, CH_2	3.18/3.96
	5	46.3, CH	3.38		NH		8.98, t (6.0)
**Cys-5**	1	170.5, C		**Tyr-14**	1	172.5, C	
	2	52.58, CH	4.56		2	52.6, CH	4.56
	3	33.5, CH_2	2.65/3.10		3	38.8, CH_2	2.27/2.69
	NH		8.52, d (8.2)		4	126.8, C	
**Abu-6**	1	171.2, C			5/9	114.8, CH	6.56, d (8.4)
	2	54.1, CH	4.30		6/8	130.5, CH	6.91, d (8.4)
	3	25.6, CH_2	1.56/1.69		7	155.8, C	
	4	10.3, CH_3	0.87, m		NH		7.47, d (9.2)
	NH		8.87, d (7.2)		OH		9.09, s
**Val-7**	1	170.5, C		**Phe-15**	1	171.6, C	
	2	57.0, CH	4.20		2	56.0, CH	4.25
	3	30.6, CH	1.96		3	35.6, CH_2	2.91
	4	17.8, CH_3	0.75, d (6.7)		4	136.9, C	
	4’	19.3, CH_3	0.81, d (6.7)		5/9	129.0, CH	7.26
	NH		7.75, d (7.8)		6/7/8	128.3, CH	7.31
**Ala-8**	1	170.5, C			NH		8.87, d (3.7)
	2	48.3, CH	4.22	**Ala-16**	1	170.8 C	
	3	18.8, CH_3	0.87, m		2	49.5, CH	4.04
	NH		7.69, d (7.8)		3	17.0, CH_3	1.17, d 97.5)
**Ala-9**	1	170.4, C			NH		8.75, d (6.5)
	2	48.30, CH	4.34				
	3	18.1, CH_3	1.15, d (7.3)				
	NH		8.11, d (7.2)				

Table S5. ^{1}H and ^{13}C NMR data of sistertide A1 (**2**) in DMSO-*d6*.

	Position	δ_C	δ_H, mult(*J* in Hz)		Position	δ_C	δ_H, mult(*J* in Hz)
**NMe$_2$-Thr-1**	1	171.9		**Ala-18**	1		
	2	16.7, CH_3	1.35, d (7.1)		2		
	3	39.9, CH	3.36		3		
	4	73.6, CH	3.64		NH		
	$N(CH_3)_2$	43.2, CH_3	2.35, s	**AviMeCys-29**	1	172.2, C	
	$N(CH_3)_2$	43.2, CH_3	2.25, s		2	55.4, CH	4.22
**Dhb-2**	1	164.1, C			3	44.3, CH	3.16
	2	130.0, C			3’	18.1, CH_3	1.20
	3	127.6, CH	6.32, q (7.2)		4	102.8, CH	5.41, d (8.4)
	4	12.7, CH_3	1.68, d (7.2)		5	122.8, CH	6.76, d (8.4)
	NH		9.26, s		5-NH		8.72
**Tyr-3**	1				NH		7.48, d (7.1)
	2	56.8, CH	3.78	**Tyr-30**	1		
	3	33.6, CH_2	2.97/3.07		2	56.9, CH	4.14
	4	128.6, C			3	35.0, CH_2	2.80/3.08
	5/9	129.9, CH	6.87, d (8.3)		4	128.2, C	
	6/8	115.0, CH	6.58, d (8.3)		5/9	129.6, CH	7.07
	7	155.8, C			6/8	115.0, CH	6.70, d (8.4)
	OH		9.27		7	155.5, C	
	NH		8.18		OH		9.08, s
**Cys-5**	1	171.6, C			NH		7.79, d (7.6)
	2	76.8, CH	3.85	**Trp-32**	1		
	3	47.9, CH_2	2.91/3.25		2	53.6, CH	4.24
	NH				3	26.2, CH_2	3.13/3.19
**Dhb-13**	1	166.8, C			4	109.1, C	
	2	132.2, C			5	127.0, C	
	3	120.9, CH	5.49		6	119.8, CH	7.54
	4	12.4, CH_3	1.71, d (6.9)		7	118.2, CH	6.98, d (7.8)
	NH		9.23		8	120.9, CH	7.06
**Cys-14**	1	170.2			9	111.3, CH	7.34, d (8.0)
	2	56.8, CH	4.15		10	136.1, C	
	3	34.3, CH_2	2.81/3.07		11	123.8, CH	7.27, d (1.8)
	NH		8.62, d (7.1)		NH (indole)		10.91, s
**Dha-15**	1	167.9, C			NH		7.83
	2	137.2, C		**Thr-33**	1	170.2, C	
	3	108.0, CH2	5.17/5.33		2	72.9, CH	
	NH		9.51		3	66.2, CH	4.41
**Val-16**	1	172.8, C			4	15.6, CH_3	1.11, d (6.4)
	2	61.7, CH	3.94				
	3	28.6. CH	2.12				
	4	18.9, CH_3	0.96, d (6.6)				
	4’	19.0, CH_3	1.00, d (6.6)				
	NH		8.91				

Table S6. ^{1}H NMR data of kebanetide A1 (**4**) in DMSO-*d6*.

	Position	δ_H, mult(*J* in Hz)		Position	δ_H, mult(*J* in Hz)
**NMe$_2$-Thr-1**	1		**AviMeCys-26**	1	
	2			2	4.49, m
	3	3.62, m		3	3.18, m
	4	1.33, d (7.0)		3’	1.21, m
	N(CH$_3$)$_2$			4	5.42, d (8.3)
	N(CH$_3$)$_2$			5	6.75, t (8.7)
**Dhb-2**	1			5-NH	8.67, brs
	2			NH	7.87, d (7.1)
	3	5.90, d (5.5)	**Tyr-27**	1	
	4	1.49, d (6.8)		2	
	NH			3	
**Trp-14**	1			4	
	2			5/9	6.88, d (8.4)
	3			6/8	6.58, d (8.1)
	5-NH	10.81, s		7	
	5	7.11, s		OH	9.03, s
	7	7.32, d (8.2)			
	8	6.96, t (7.5)			
	9	7.05, t (7.5)			
	10	7.53, d (8.2)			

Table S7. ^{1}H NMR data of kebanetide A2 (**5**) in DMSO-*d6*.

	**Position**	**δ_H, mult(*J* in Hz)**
**Dhb-10**	1	
	2	
	3	6.39, q (7.4)
	4	1.60, d (6.8)
	NH	
**AviMeCys-22**	1	
	2	
	3	
	3'	
	4	5.16, m
	5	6.20, dd (5.84,2.05)
	5-NH	7.85, brs
	NH	

Table S8. Advanced Marfey's analysis for massatide (**1**).

residue	derivative with L-FDLA (retention time/min)	derivative with D-FDLA (retention time/min)	Configuration
Ala	15.33, 16.49	15.60, 16.72	L, D
Abu	17.44	16.09	D
Val	16.31	18.58	L
Pro	15.30	16.42	L
Phe	17.23	19.10	L
Tyr	19.95	21.17	L
*D-Abu std.	17.67	16.24	

Table S9. Advanced Marfey's analysis for sistertide A1 (**2**).

residue	derivative with L-FDLA (retention time/min)	derivative with D-FDLA (retention time/min)	Configuration
Ala	15.35, 16.51	15.59, 16.71	L, D
Val	16.32	18.58	L
Ile	17.06	19.58	L
Arg	12.75	12.48	L
Lys (di)	19.61	20.36	L
Glu	14.58	15.14	L
Thr	14.08	15.68	L
Tyr (di)	19.95	21.19	L
Trp	n.d	n.d	

*n. d. Not detected

Table S10. Advanced Marfey's analysis for kebanetide A1 (**4**).

residue	derivative with L-FDLA (retention time/min)	derivative with D-FDLA (retention time/min)	Configuration
Ala	15.38, 16.46	15.57, 16.65	L, D
Val	16.30	18.51	L
Leu	17.16	19.62	L
Pro	15.31	16.37	L
Glu	14.61	15.13	L
Thr	14.09	15.64	L
Tyr (di)	19.92	21.11	L
Trp	n.d	n.d	

n. d. Not detected

Table S11. Advanced Marfey's analysis for kebanetide A2 (**5**).

residue	derivative with L-FDLA (retention time/min)	derivative with D-FDLA (retention time/min)	Configuration
Ala	15.31, 16.42	15.55, 16.64	L, D
Val	16.26	18.52	L
Ile	16.99	19.62	L
Pro	15.26	16.37	L
Lys (di)	19.58	20.29	L
Gln*	14.54	15.12	L
Phe	17.19	19.04	L

* Gln convert to Glu in acid hydrolysis

Supplementary figures

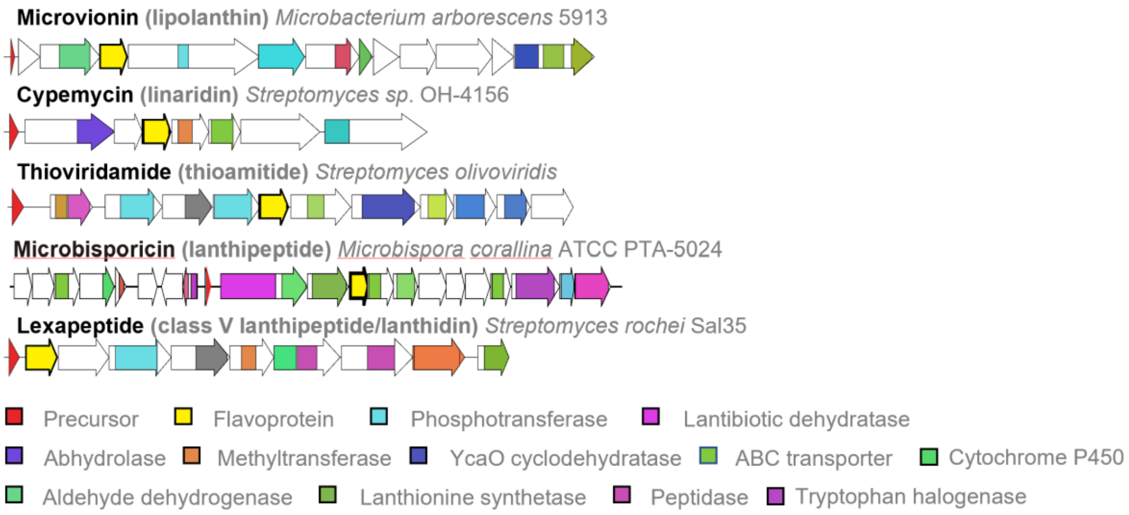

Figure S1. Biosynthetic gene clusters of known Avi(Me)Cys-containing RiPPs.

A

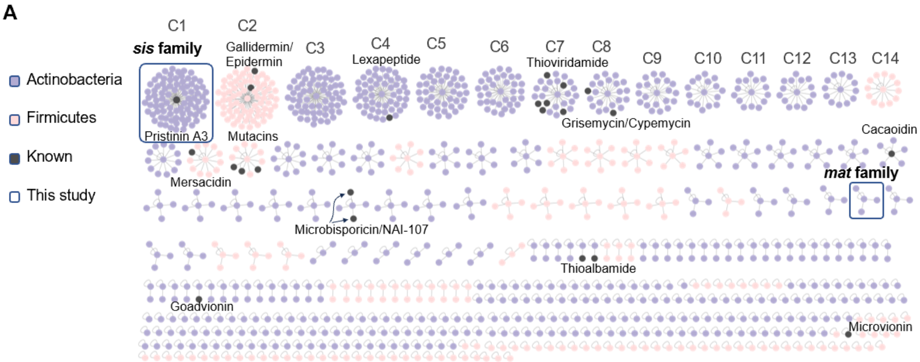

B

Precursor logos and BGC phylogenetic analysis, representative clade was shown below

Cluster C1, the *sis* family

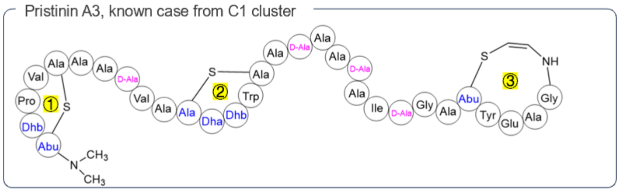

Chemical diversity: BGCs from C1 cluster contain multiple precursors. Precursors with both 2 cysteine residues and 3 cysteine residues are found in these BGCs, implying the encoded products may contain 2 or 3 thioether bridges. Variations inside the AviCys and thioether rings largely expanded the chemical diversity.

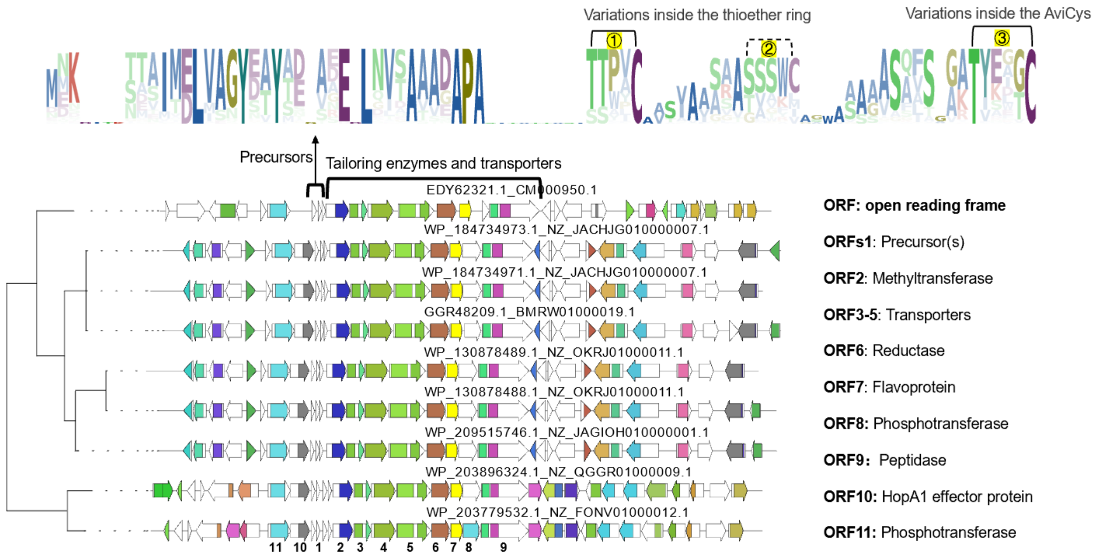

(Figure S2 continues)

C **Cluster C2**

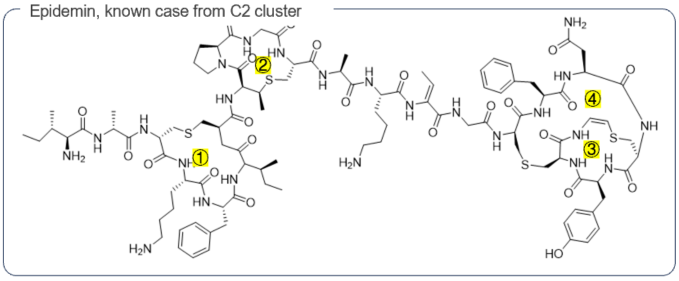

Chemical diversity: BGCs from C2 cluster contain one or two precursors. The putative rings were highlighted below. Variations inside the thioether ring 1 largely expanded the chemical diversity.

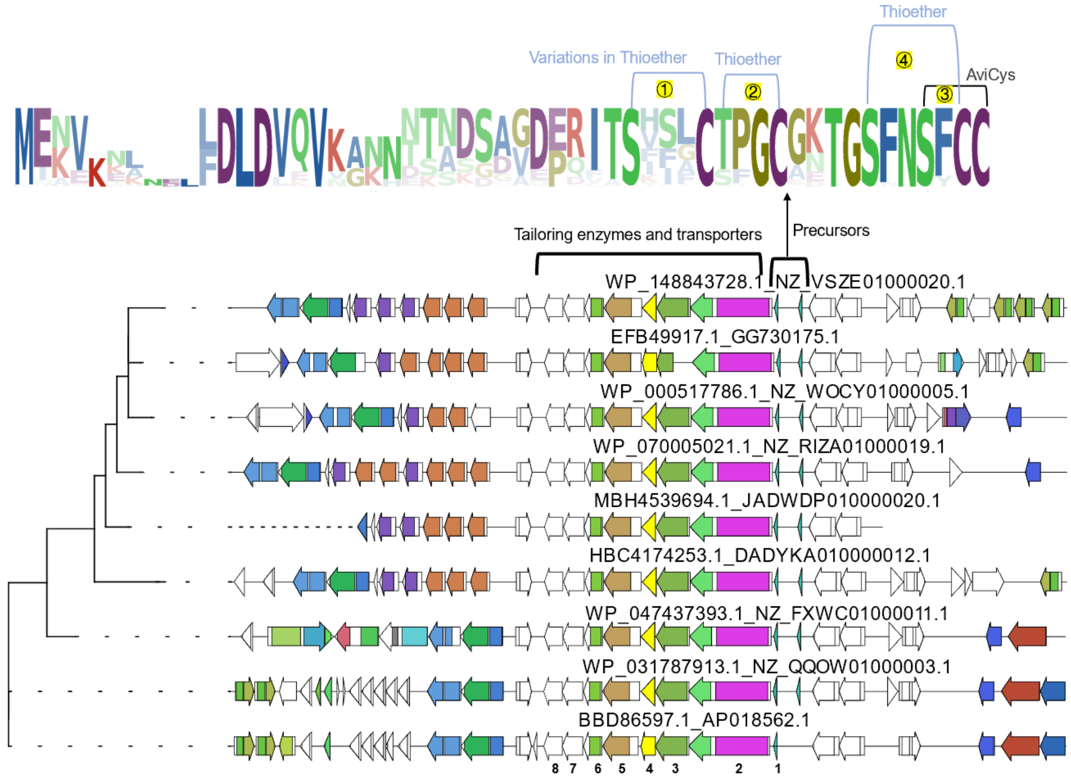

ORF: open reading frame | **ORFs1**: Precursor(s)

ORF2: Lantibiotic dehydratase C-terminus | **ORF3**: Lanthionine synthease

ORF4: Flavoprotein Reductase | **ORF5**: Peptidase

ORF6: Transporters | **ORF7**: Unknown function | **ORF8:** Unknown function

(Figure S2 continues)

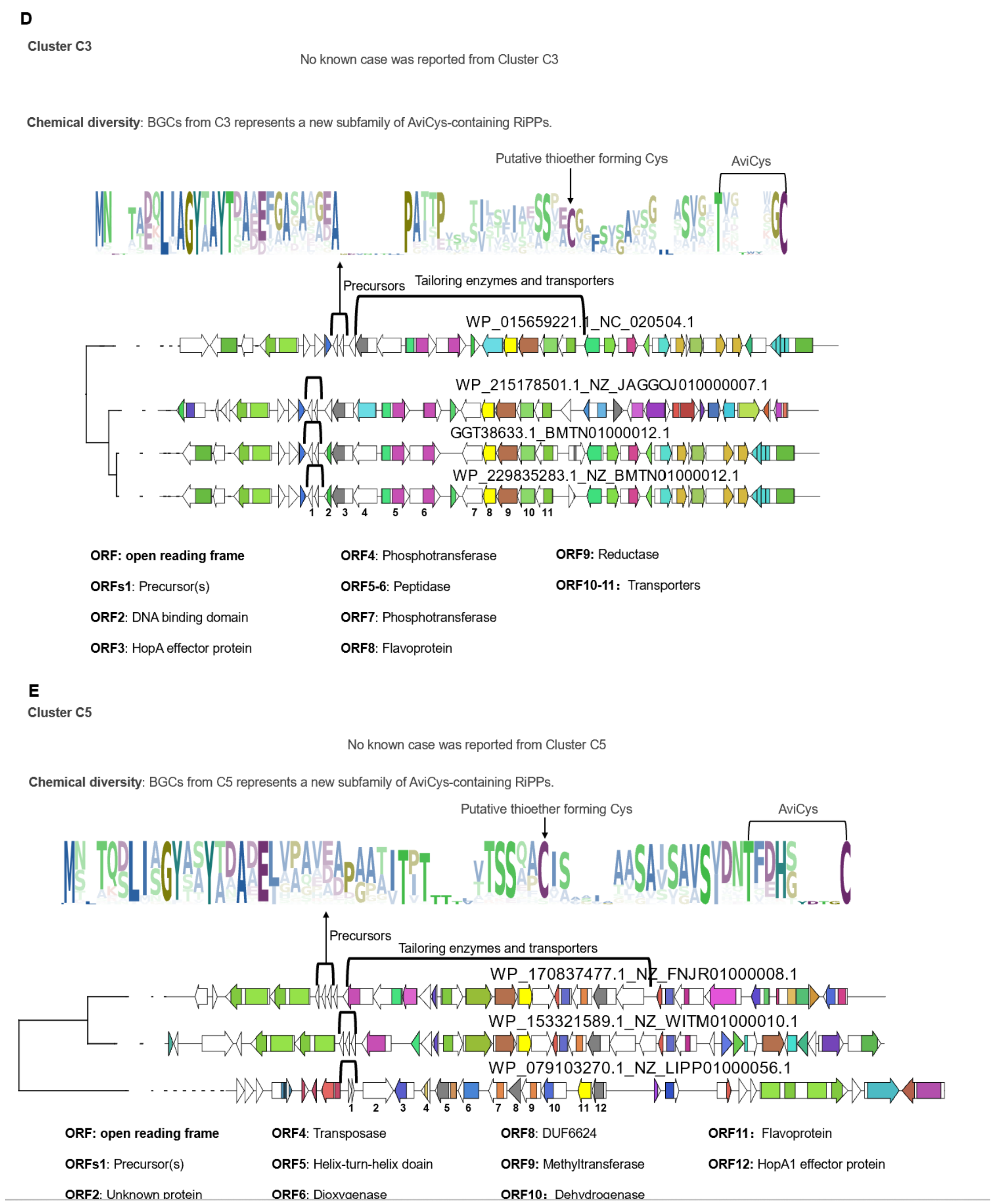

Figure S2. Expanded cluster in precursor SSN.

A. SSN of the precursors. B. Sequence logo and BGC phylogenetic analysis of cluster 1, *sis* family. C. Sequence logo and BGC phylogenetic analysis of cluster 2. D. Sequence logo and BGC phylogenetic analysis of cluster 3. E. Sequence logo and BGC phylogenetic analysis of cluster 5.

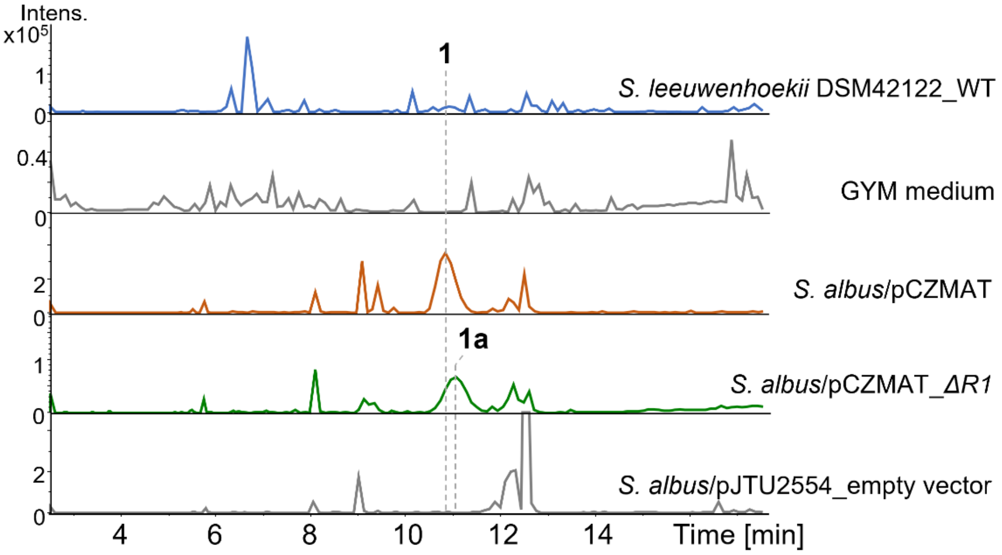

Figure S3. UPLC-HRMS analysis of DSM42122 wild-type strain and heterologous expression of *mat* BGC.
Source data are provided in MassIVE (DOI: 10.25345/C5PC2TM3Q).

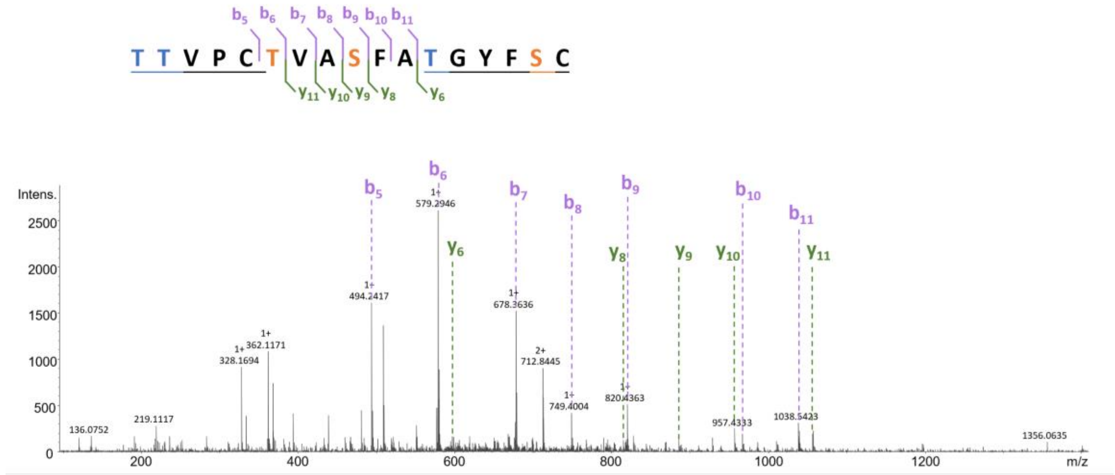

ions	modification	z	observed m/z	calculated m/z	Δ ppm
b5	$-2H_2O\_+2CH_2$	1	494.2417	494.2432	3.0
b6	$-3H_2O\_+2CH_2\_+2H$	1	579.2946	579.2959	2.2
b7	$-3H_2O\_+2CH_2\_+2H$	1	678.3636	678.3643	1.0
b8	$-3H_2O\_+2CH_2\_+2H$	1	749.4004	749.4015	1.5
b9	$-4H_2O\_+2CH_2\_+4H$	1	820.4363	820.4386	2.8
b10	$-4H_2O\_+2CH_2\_+4H$	1	967.5063	967.5070	0.7
b11	$-4H_2O\_+2CH_2\_+4H$	1	1038.5423	1038.5441	1.7
y6	$-2H_2O\_-1CO_2H_2\_+2H$	1	597.2483	597.2495	2.0
y8	$-2H_2O\_-1CO_2H_2\_+2H$	1	815.3562	815.3551	1.3
y9	$-3H_2O\_-1CO_2H_2\_+4H$	1	886.3907	886.3922	1.7
y10	$-3H_2O\_-1CO2H_2\_+4H$	1	957.4333	957.4293	4.2
y11	$-3H_2O\_-1CO_2H_2\_+4H$	1	1056.4962	1056.4977	1.4

Figure S4. Tandem MS/MS analysis for massatide A (**1**).
Residues in blue represent the amino acid that is dehydrated ($-H_2O$). Residues in orange represent the amino acid that is first dehydrated ($-H_2O$) and then hydrogenated (+2H). Underlines represent the cyclization part. Source data are provided in MassIVE (DOI: 10.25345/C5PC2TM3Q).

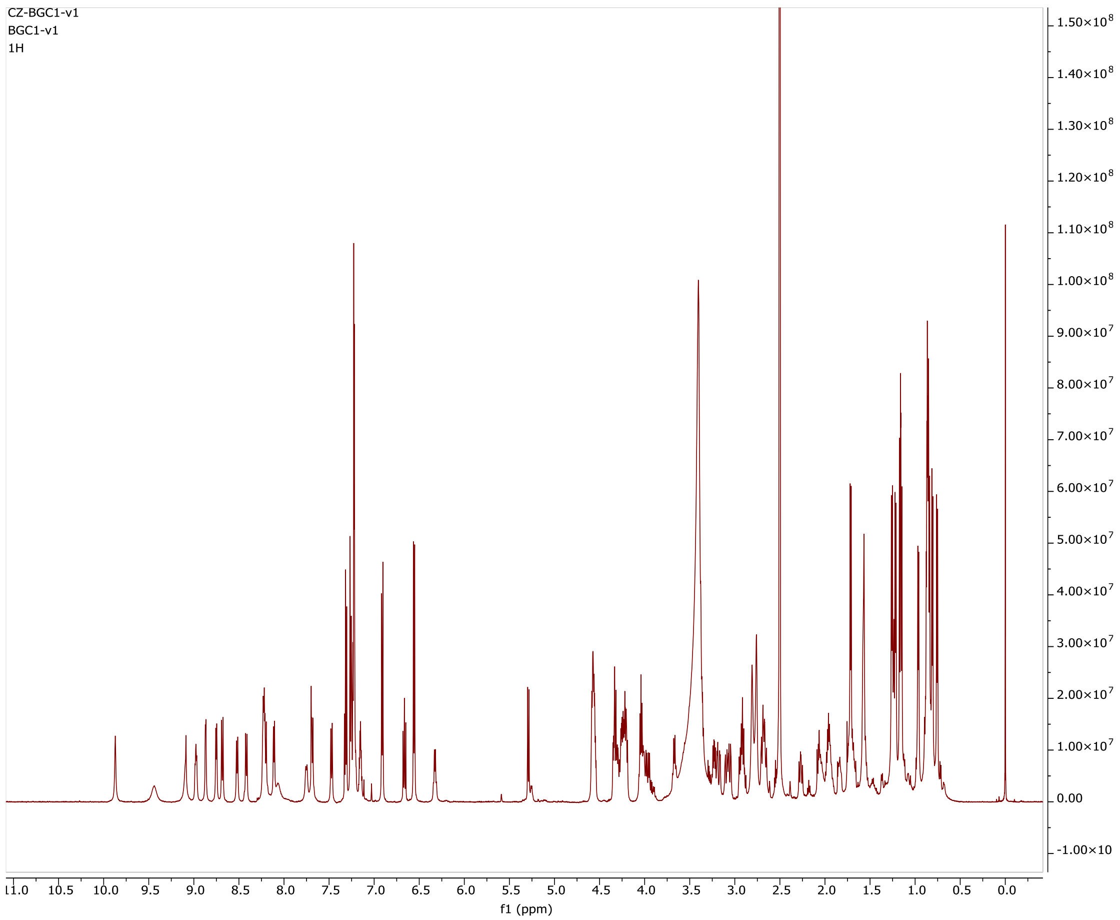

Figure S5. ^{1}H NMR spectrum (600 MHz, DMSO-*d6*) of massatideA (**1**).

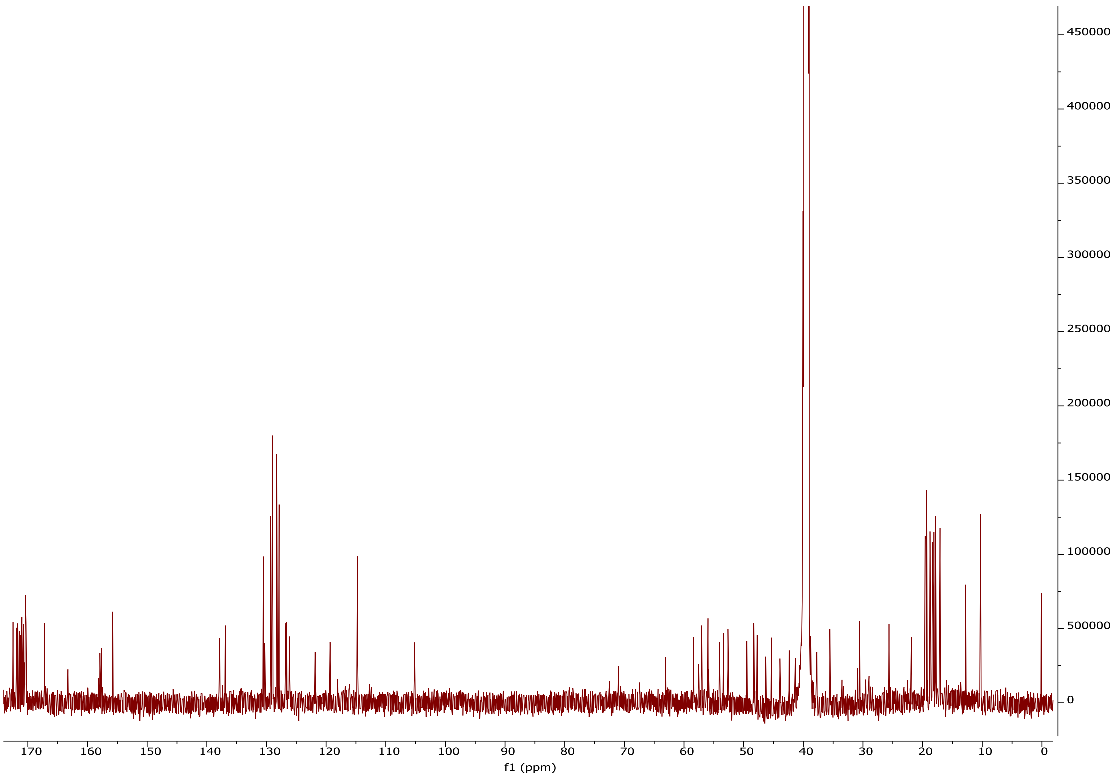

Figure S6. ^{13}C NMR spectrum (150 MHz, DMSO-*d6*) of massatide A (**1**).

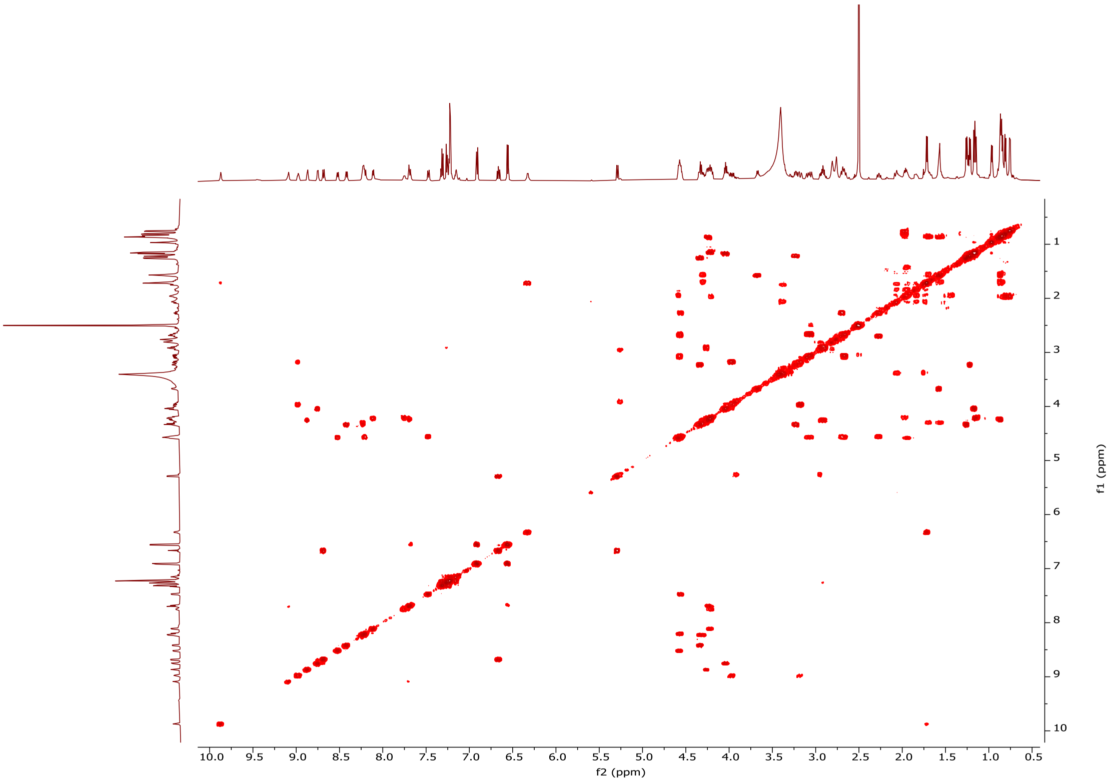

Figure S7. ^1H-^1H COSY spectrum (600 MHz, DMSO-*d6*) of massatide A (**1**).

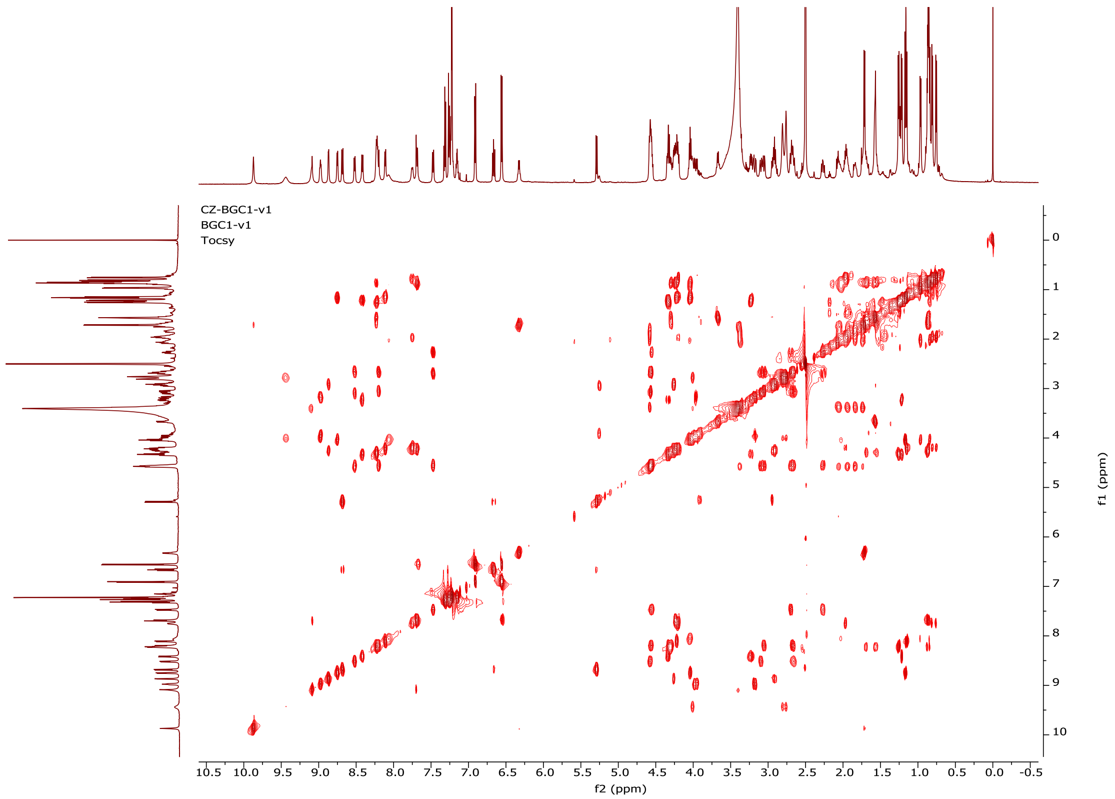

Figure S8. TOCSY spectrum (600 MHz, DMSO-*d6*) of massatide A (**1**).

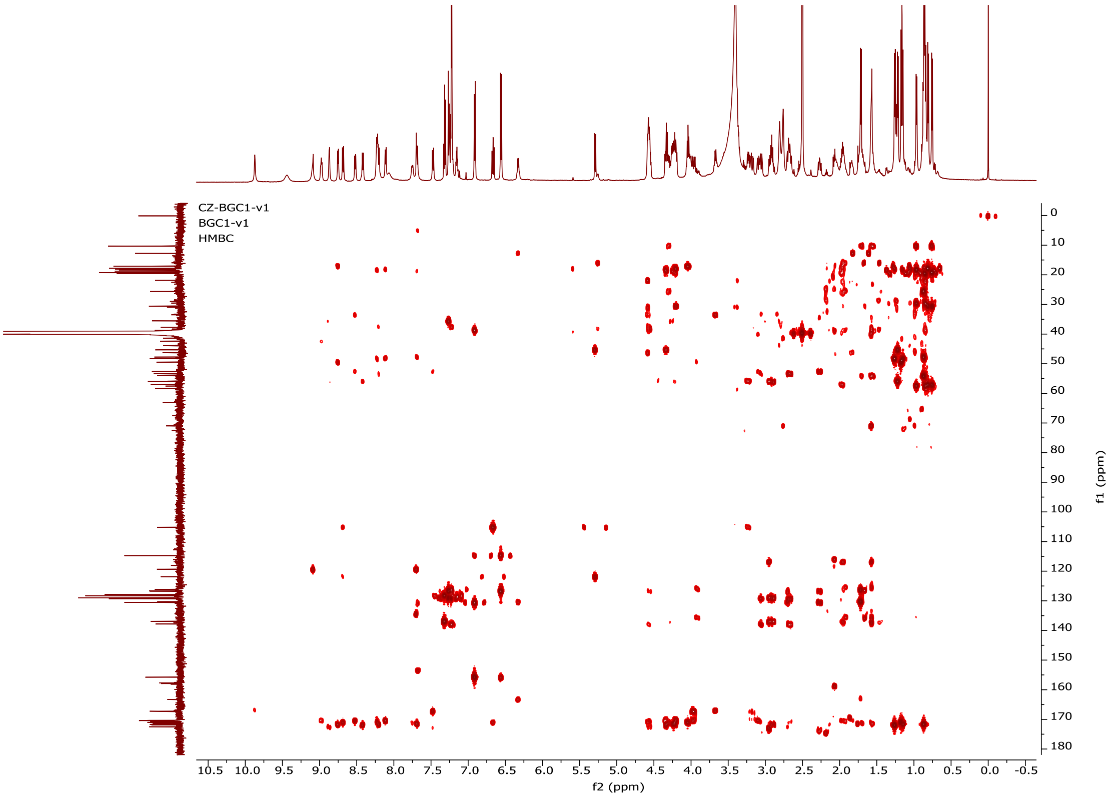

Figure S9. HMBC spectrum (600 MHz, DMSO-*d6*) of massatide A (**1**).

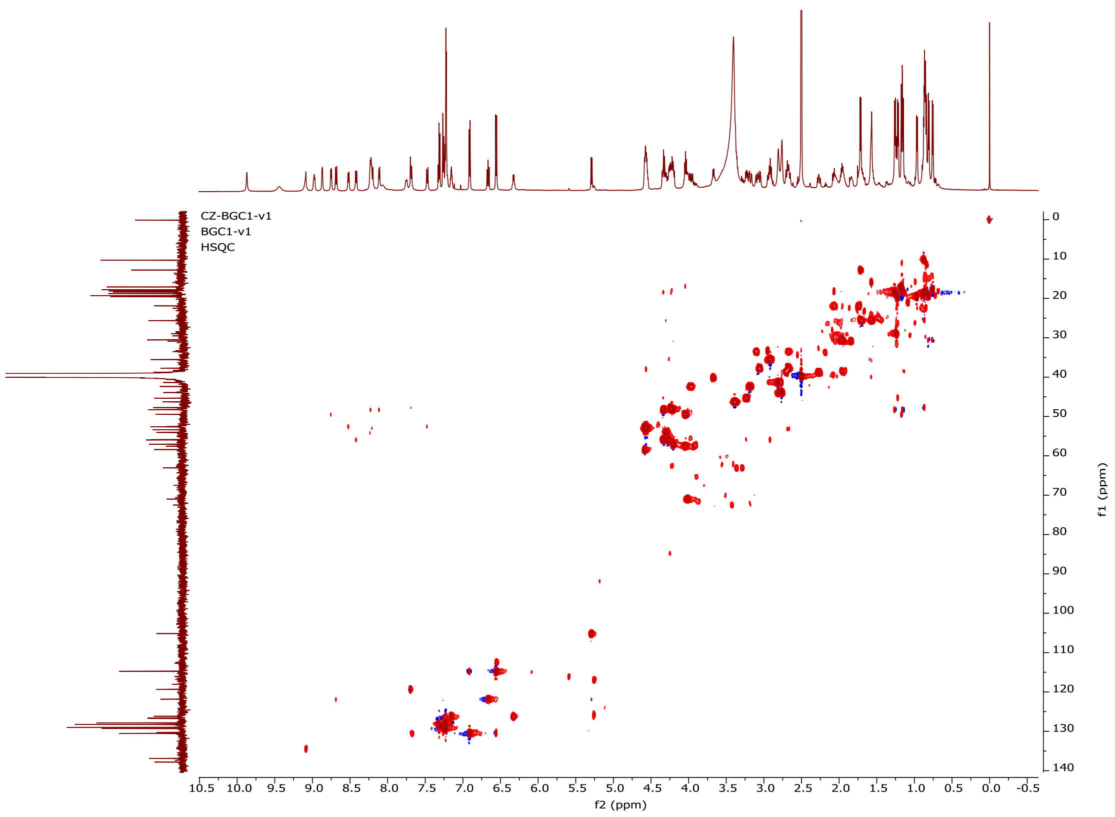

Figure S10. HSQC spectrum (600 MHz, DMSO-*d6*) of massatide A (**1**).

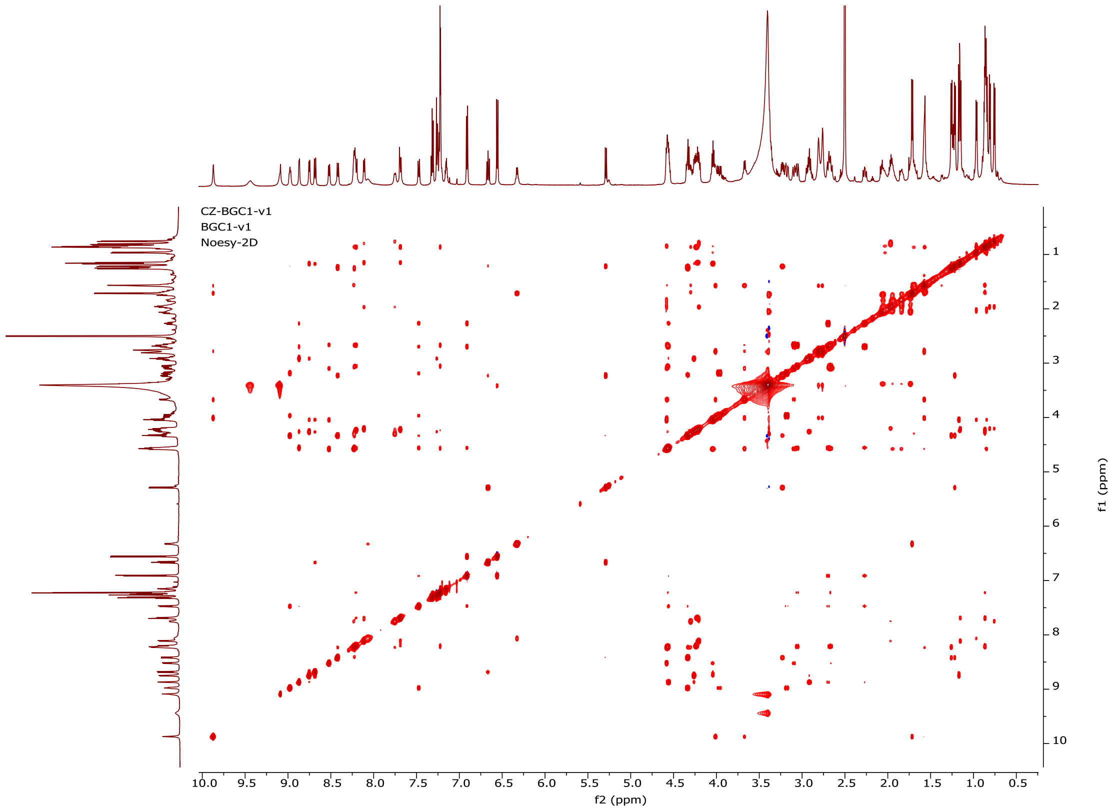

Figure S11. NOESY spectrum (600 MHz, DMSO-*d6*) of massatide A (**1**).

A

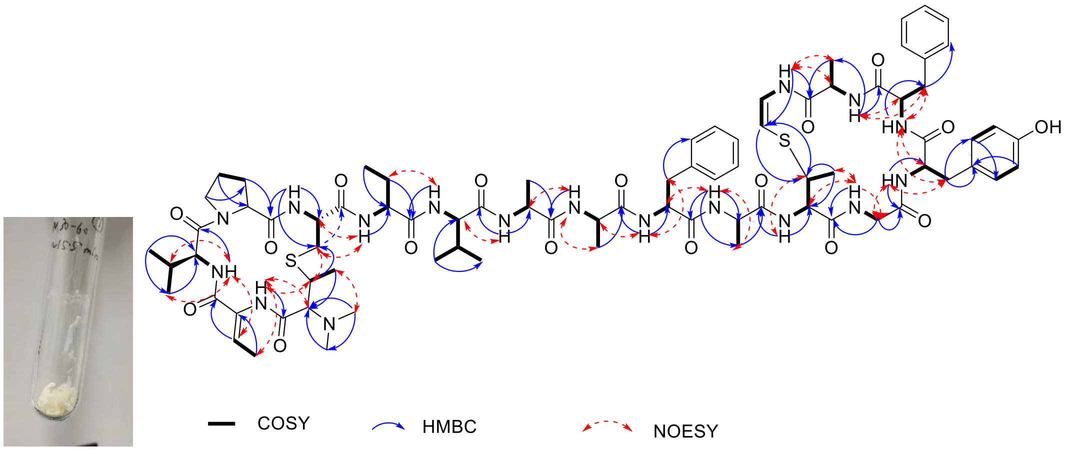

B

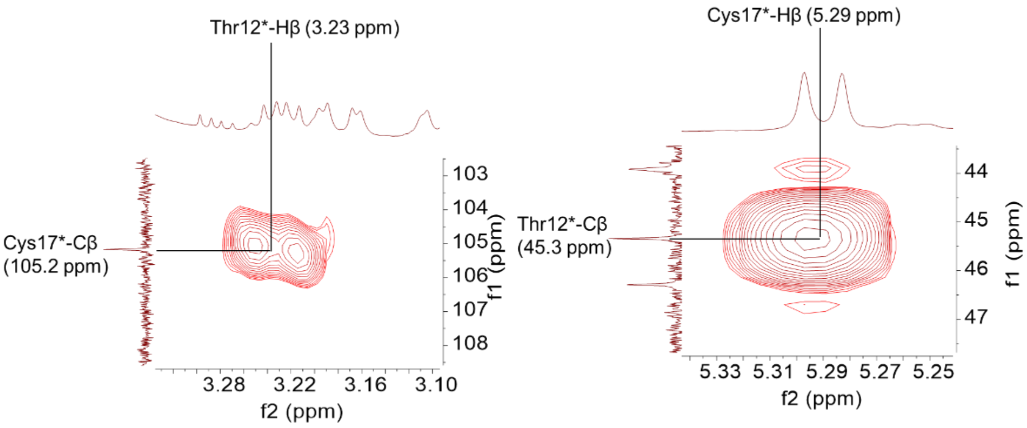

Figure S12. NMR analysis of massatide A.
A. Purified compound **1** and key COSY, HMBC, and NOESY correlations of massatide A (**1**). B**.** Key HMBC correlations.

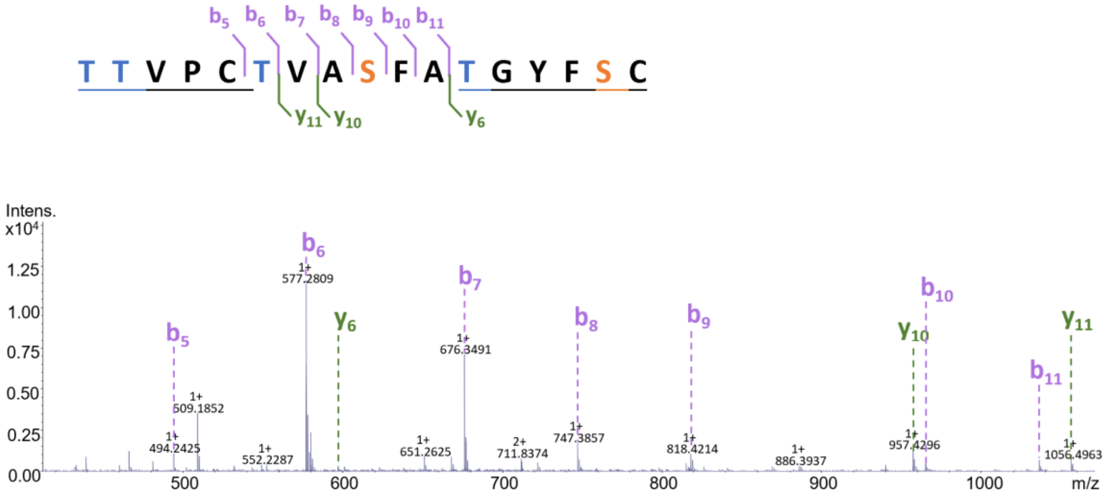

ions	modification	z	observed m/z	calculated m/z	ppm
b5	$-2H_2O\_+2CH_2$	1	494.2425	494.2432	1.4
b6	$-3H_2O\_+2CH_2$	1	577.2809	577.2803	1.1
b7	$-3H_2O\_+2CH_2$	1	676.3491	676.3487	0.6
b8	$-3H_2O\_+2CH_2$	1	747.3857	747.3858	0.1
b9	$-4H_2O\_+2CH_2\_+2H$	1	818.4214	818.4229	1.9
b10	$-4H_2O\_+2CH_2\_+2H$	1	965.4888	965.4913	2.6
b11	$-4H_2O\_+2CH_2\_+2H$	1	1036.5311	1036.5284	2.6
y6	$-2H_2O\_-CO_2H_2\_+2H$	1	597.2485	597.2495	1.7
y10	$-3H_2O\_-CO_2H_2\_+4H$	1	957.4296	957.4293	0.3
y11	$-3H_2O\_-CO_2H_2\_+4H$	1	1056.4963	1056.4977	1.3

Figure S13. Tandem MS/MS analysis for massatide B (**1a**).
Residues in blue represent the amino acid is dehydrated ($-H_2O$). Residues in orange represent the amino acid is first dehydrated ($-H_2O$) and then hydrogenated (+2H). Underlines represent the cyclization part. Source data are provided in MassIVE (DOI: 10.25345/C5PC2TM3Q).

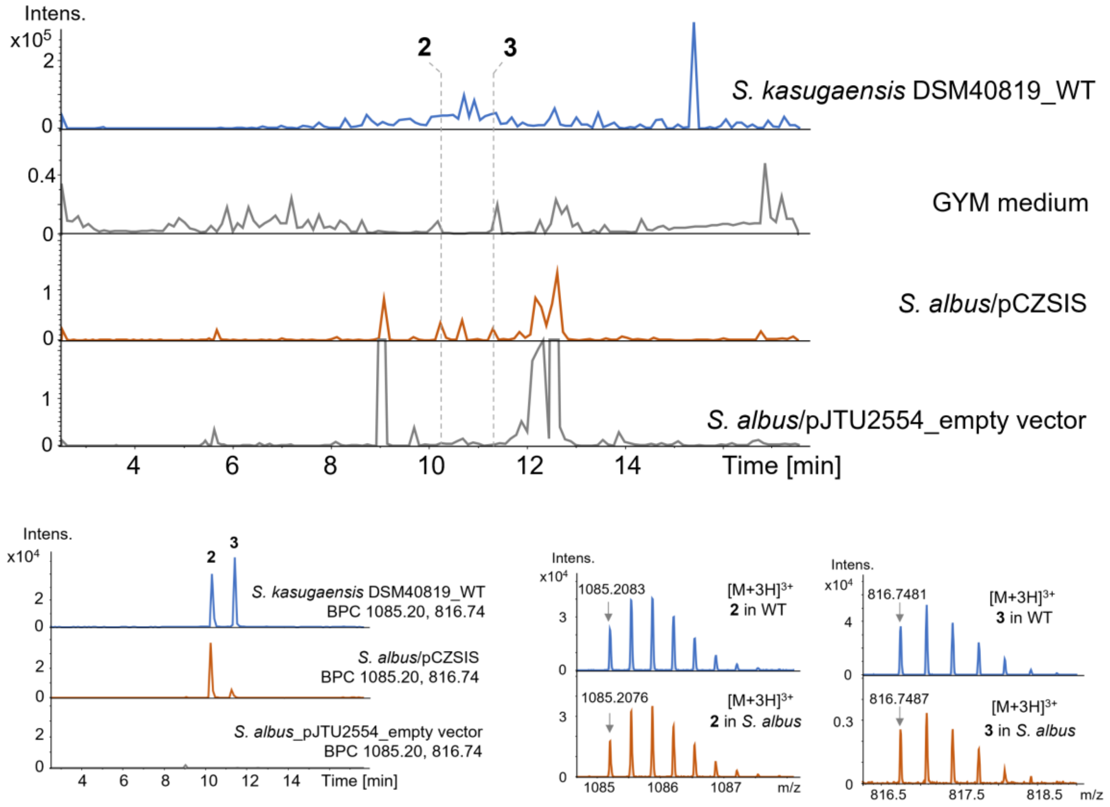

Figure S14. UPLC-HRMS analysis of DSM40819 wild-type strain and heterologous expression of *sis* BGC.
Source data are provided in MassIVE (DOI: 10.25345/C5PC2TM3Q).

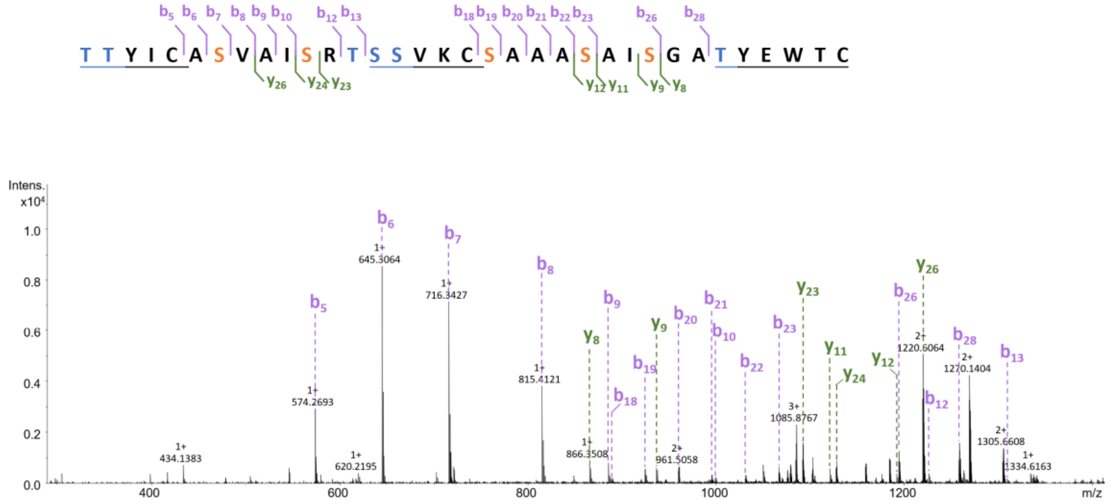

ions	modification	z	observed m/z	calculated m/z	ppm
b5	-2H_2O	1	574.2693	574.2694	0.1
b6	-2H_2O	1	645.3064	645.3065	0.1
b7	-3H_2O_+2CH_2_+2H	1	716.3427	716.3436	1.3
b8	-3H_2O_+2CH_2_+2H	1	815.4121	815.4120	0.1
b9	-3H_2O_+2CH_2_+2H	1	886.4479	886.4491	1.4
b10	-3H_2O_+2CH_2_+2H	1	999.5330	999.5332	0.2
b12	-4H_2O_+2CH_2_+4H	1	1226.6750	1226.6714	2.9
b13	-5H_2O_+2CH_2_+4H	1	1309.7074	1309.7085	0.9
b18	-7H_2O_+2CH_2_+4H	2	889.4592	889.4657	7.3
b19	-8H_2O_+2CH_2_+6H	2	924.9814	924.9842	3.0
b20	-8H_2O_+2CH_2_+6H	2	960.5067	960.5028	4.1
b21	-8H_2O_+2CH_2_+6H	2	996.0177	996.0213	3.6
b22	-8H_2O_+2CH_2_+6H	2	1031.5410	1031.5399	1.1
b23	-9H_2O_+2CH_2_+8H	2	1067.0564	1067.0584	1.9
b26	-10H_2O_+2CH_2_+10H	2	1194.6398	1194.6376	1.9
b28	-10H_2O_+2CH_2_+10H	2	1258.6620	1258.6669	3.9
y8	-1H_2O_-1CO_2H_2	1	866.3508	866.3507	0.1
y9	-2H_2O_-1CO_2H_2_+2H	1	937.3861	937.3878	1.8
y11	-2H_2O_-1CO_2H_2_+2H	1	1121.5054	1121.5090	3.2
y12	-3H_2O_-1CO_2H_2_+4H	1	1192.5404	1192.5461	4.8
y23	-7H_2O_-1CO_2H_2_+6H	2	1092.5262	1092.5278	1.4
y24	-8H_2O_-1CO_2H_2_+8H	2	1128.0416	1128.0463	4.2
y26	-8H_2O_-1CO_2H_2_+8H	2	1220.1070	1220.1069	0.1

Figure S15. Tandem MS/MS analysis for sistertide A1 (**2**).
Residues in blue represent the amino acid is dehydrated (-H_2O). Residues in orange represent the amino acid is first dehydrated (-H_2O) and then hydrogenated (+2H). Underlines represent the cyclization part. Source data are provided in MassIVE (DOI: 10.25345/C5PC2TM3Q).

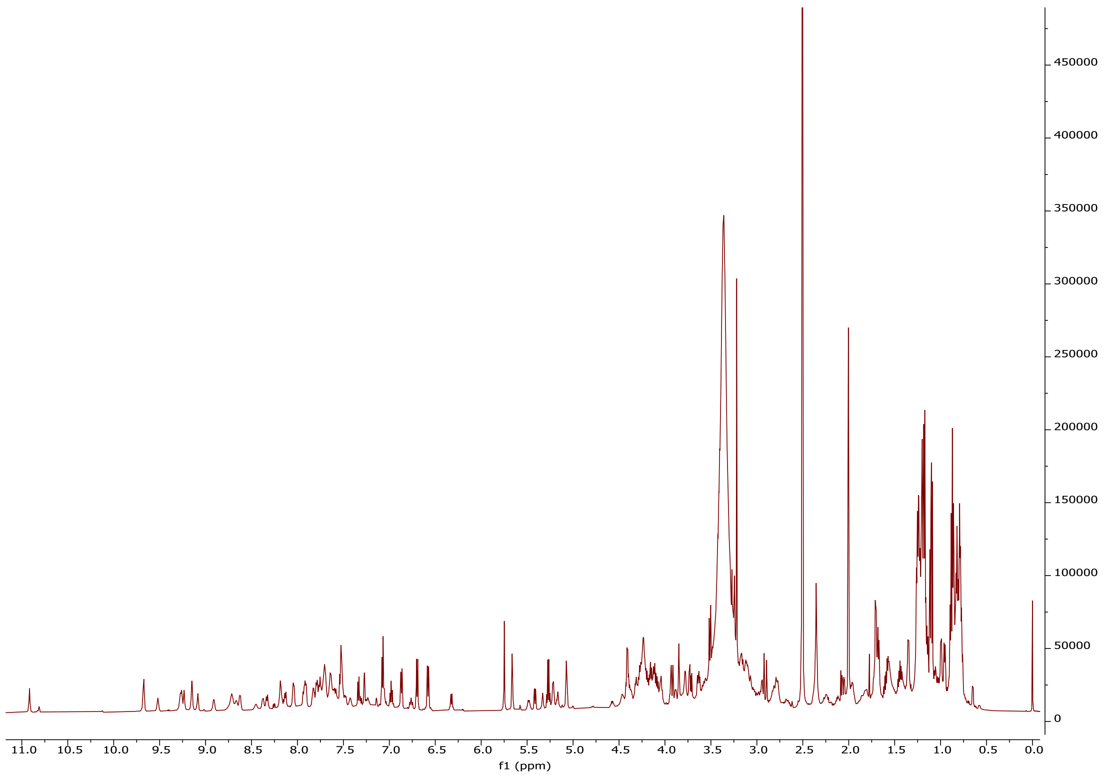

Figure S16. ^{1}H NMR spectrum (600 MHz, DMSO-*d6*) of sistertide A1 (**2**).

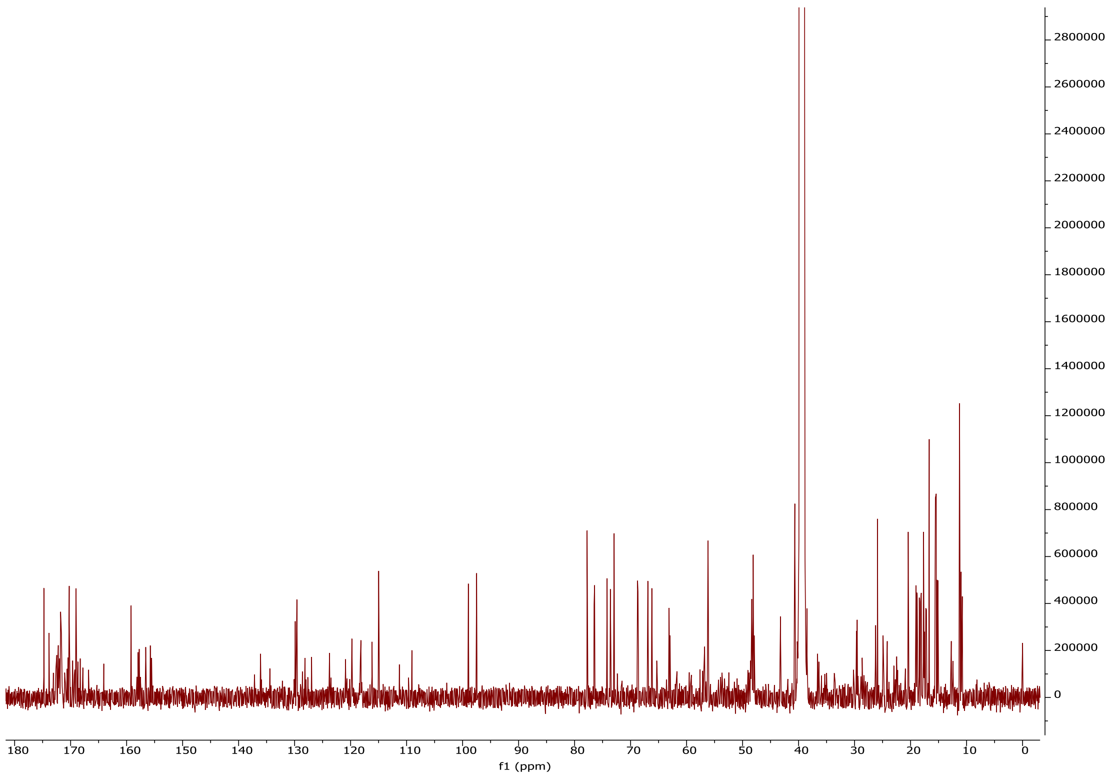

Figure S17. ^{13}C NMR spectrum (150 MHz, DMSO-*d6*) of sistertide A1 (**2**).

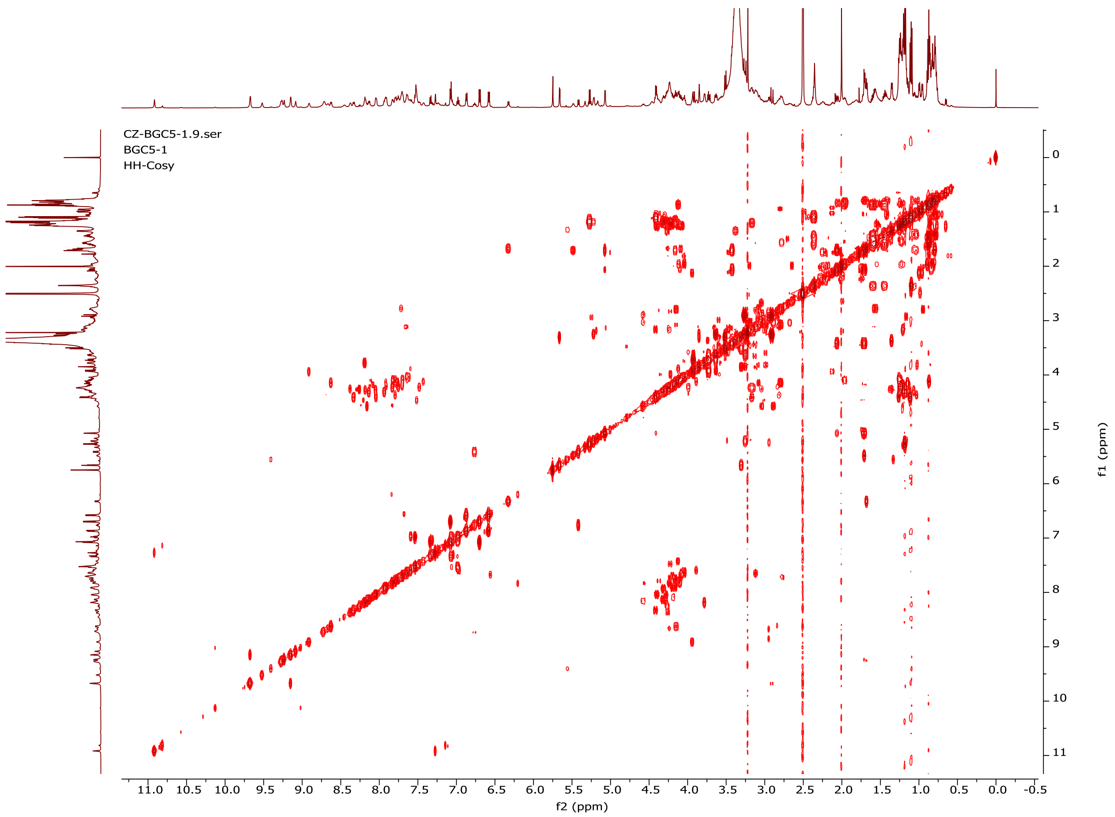

Figure S18. ^{1}H-^{1}H COSY spectrum (600 MHz, DMSO-*d6*) of sistertide A1 (**2**).

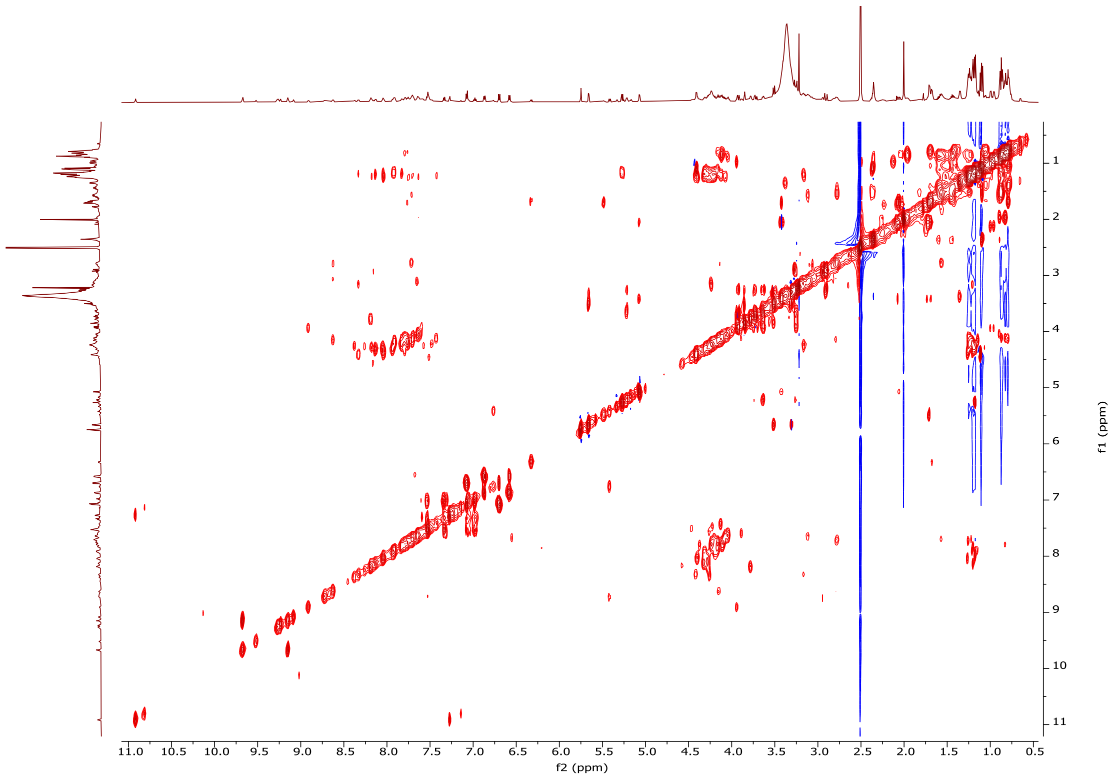

Figure S19. TOCSY spectrum (600 MHz, DMSO-*d6*) of sistertide A1 (**2**).

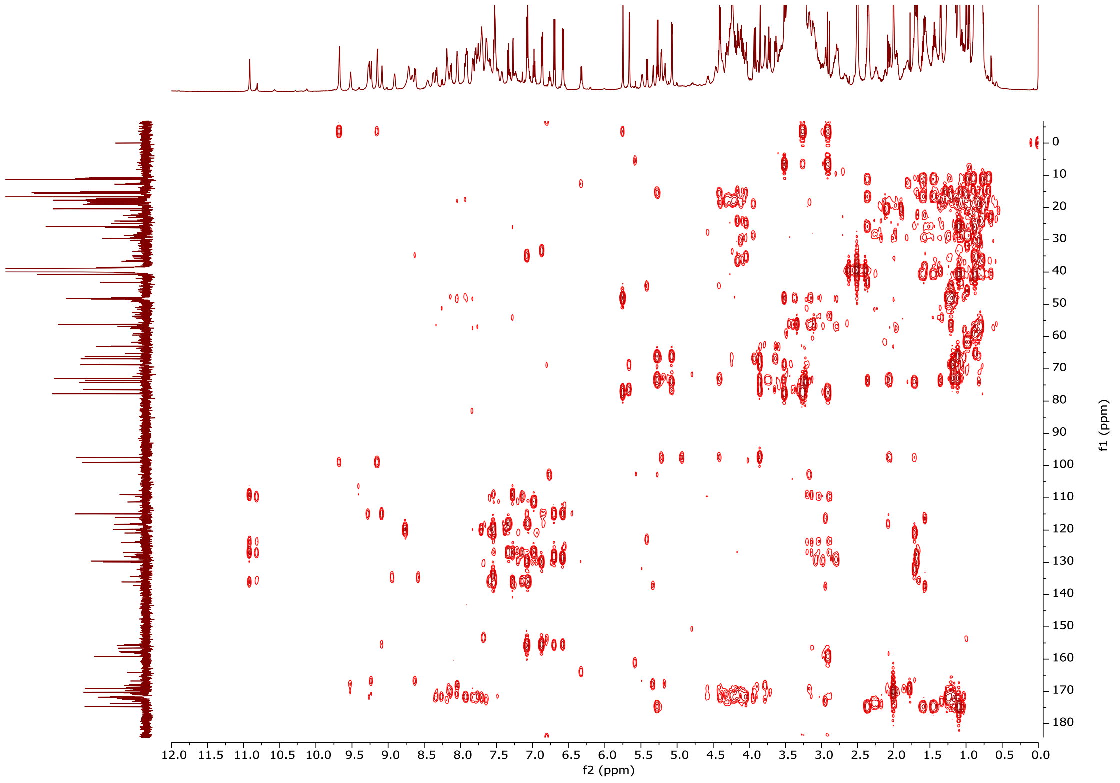

Figure S20. HMBC spectrum (600 MHz, DMSO-*d6*) of sistertide A1 (**2**).

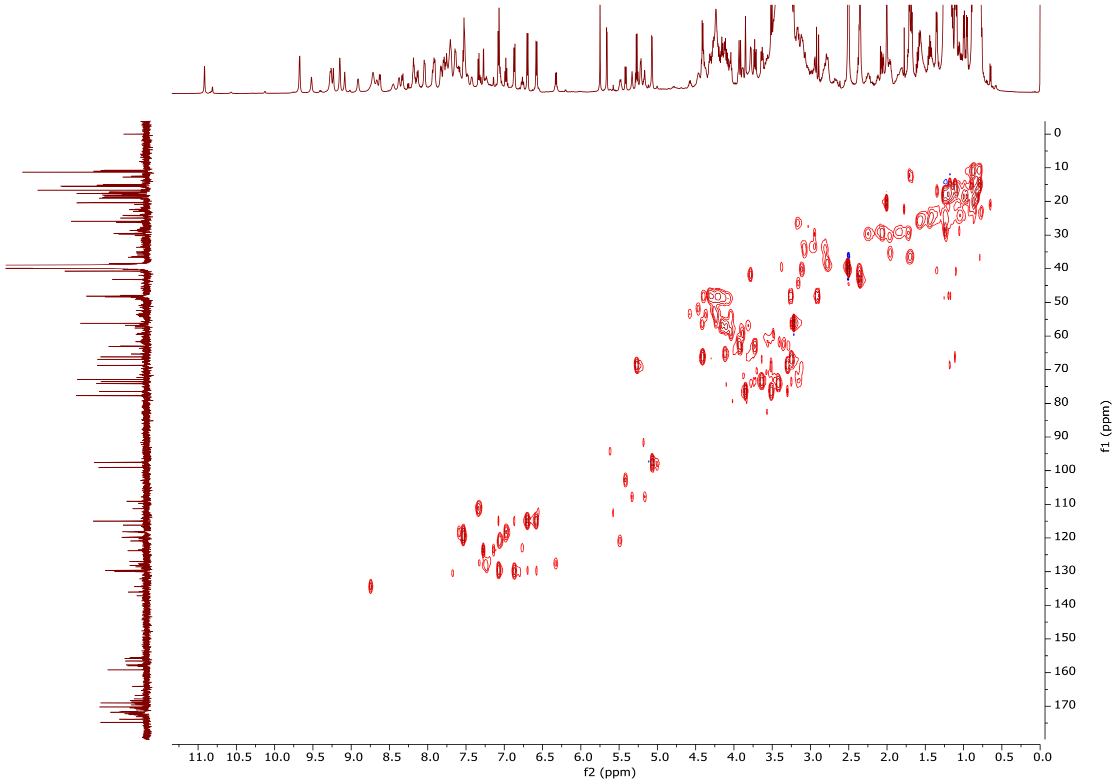

Figure S21. HSQC spectrum (600 MHz, DMSO-*d6*) of sistertide A1 (**2**).

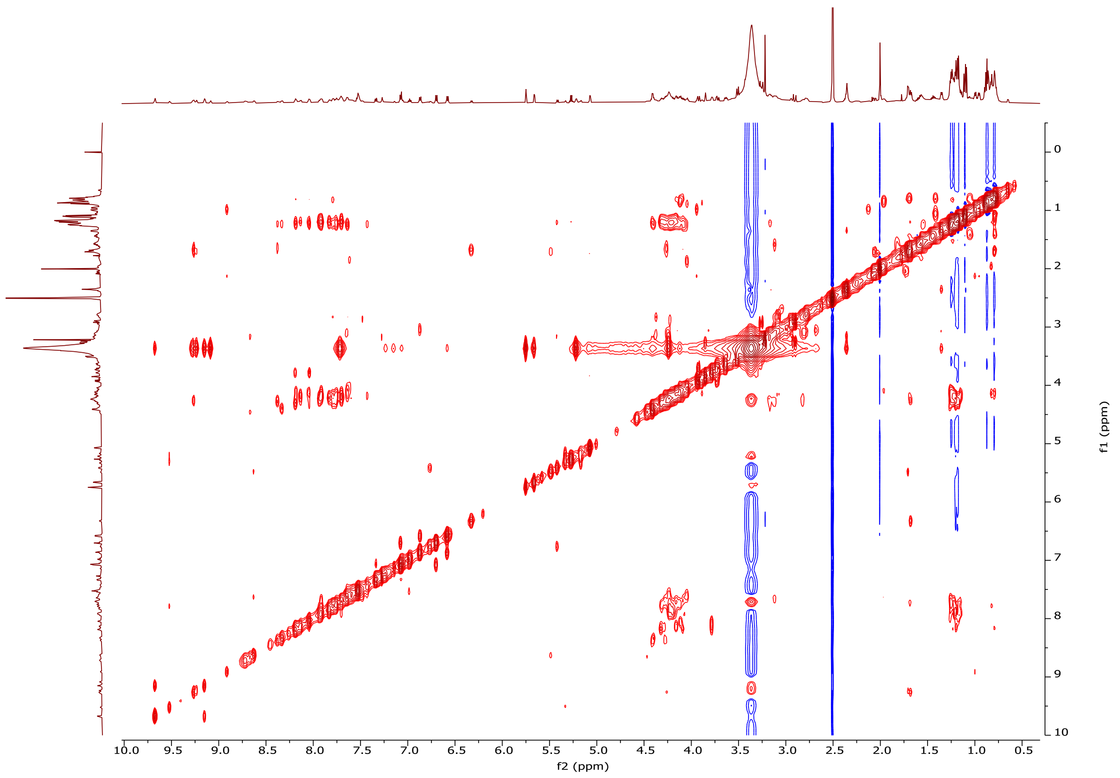

Figure S22. NOESY spectrum (600 MHz, DMSO-*d6*) of sistertide A1 (**2**).

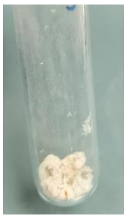

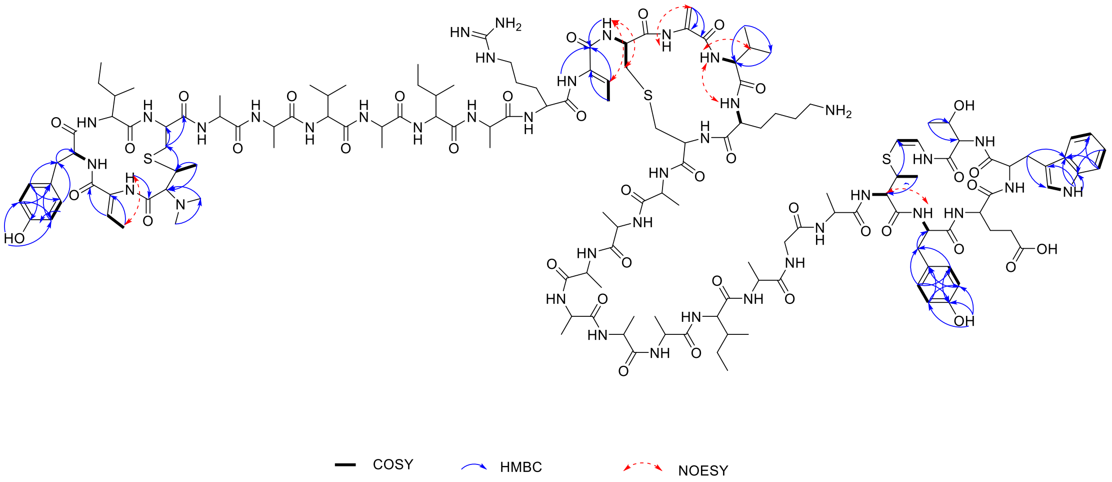

Figure S23. Purified compound **2**. Key COSY, HMBC, and NOESY correlations of sistertide A1 (**2**).

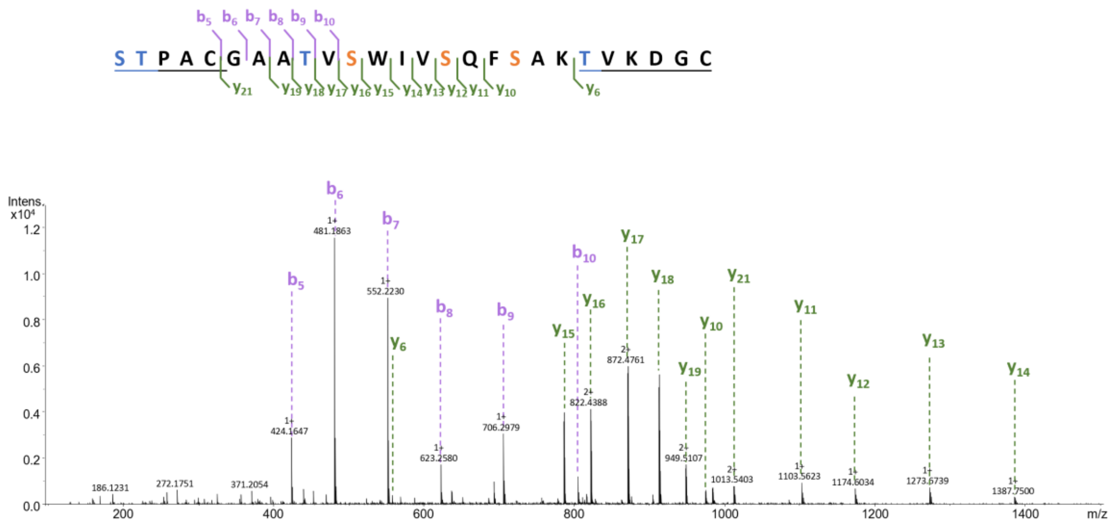

ions	modification	z	observed m/z	calculated m/z	Δ ppm
b5	$-2H_2O$	1	424.1647	424.1649	0.5
b6	$-2H_2O$	1	481.1863	481.1864	0.2
b7	$-2H_2O$	1	552.2230	552.2235	0.9
b8	$-2H_2O$	1	623.2580	623.2606	4.2
b9	$-3H_2O$	1	706.2979	706.2977	0.3
b10	$-3H_2O$	1	805.3646	805.3661	1.9
y6	$-1H_2O\_-1CO_2H_2$	1	558.2699	558.2710	2.0
y10	$-2H_2O\_-1CO_2H_2\_+2H$	1	975.5065	975.5086	2.1
y11	$-2H_2O\_-1CO_2H_2\_+2H$	1	1103.5623	1103.5672	4.4
y12	$-3H_2O\_-1CO_2H_2\_+4H$	1	1174.6034	1174.6043	0.8
y13	$-3H_2O\_-1CO_2H_2\_+4H$	1	1273.6739	1273.6727	0.9
y14	$-3H_2O\_-1CO_2H_2\_+4H$	1	1386.7595	1386.7568	2.0
y15	$-3H_2O\_-1CO_2H_2\_+4H$	2	786.9208	786.9217	1.1
y16	$-4H_2O\_-1CO_2H_2\_+6H$	2	822.4388	822.4402	1.7
y17	$-4H_2O\_-1CO_2H_2\_+6H$	2	871.9740	871.9744	0.5
y18	$-5H_2O\_-1CO_2H_2\_+6H$	2	913.4914	913.4930	1.8
y19	$-5H_2O\_-1CO_2H_2\_+6H$	2	949.0110	949.0116	0.6
y21	$-5H_2O\_-1CO_2H_2\_+6H$	2	1013.0406	1013.0408	0.2

Figure S24. Tandem MS/MS analysis for sistertide A2 (**3**).
Source data are provided in MassIVE (DOI: 10.25345/C5PC2TM3Q).

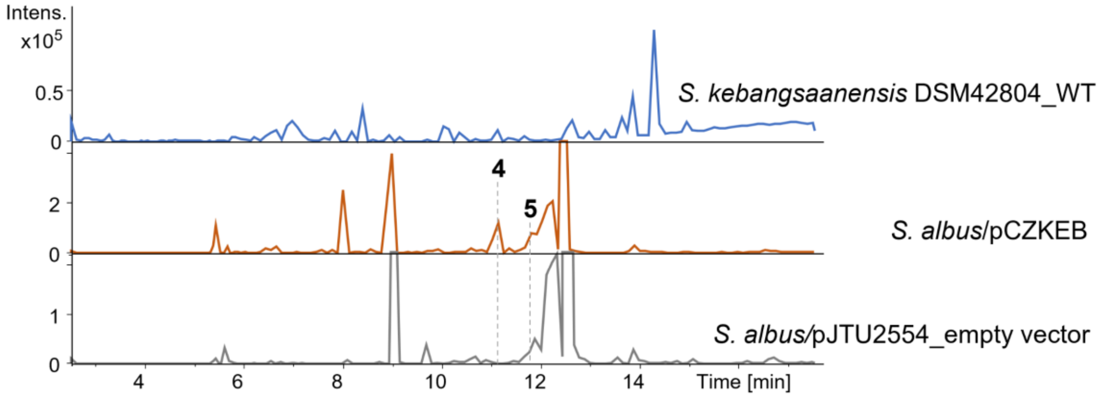

Figure S25. UPLC-HRMS analysis of DSM 42048 wild type strain and heterologous expression of *keb* BGC.
Source data are provided in MassIVE (DOI: 10.25345/C5PC2TM3Q).

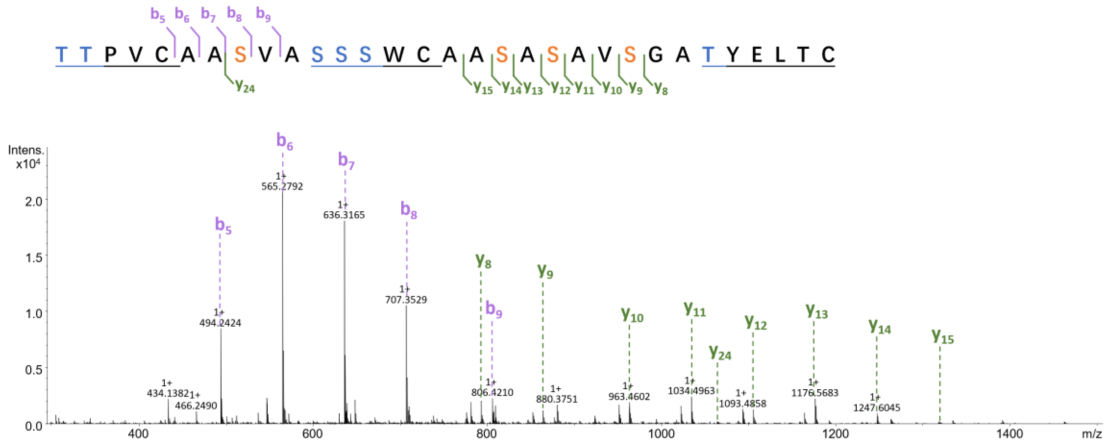

ions	modification	z	observed m/z	calculated m/z	Δ ppm
b5	-2H_2O_+2CH_2	1	494.2424	494.2432	1.6
b6	-2H_2O_+2CH_2	1	565.2792	565.2802	1.9
b7	-2H_2O_+2CH_2	1	636.3165	636.3174	1.4
b8	-3H_2O_+2CH_2+2H	1	707.3529	707.3545	2.3
b9	-3H_2O_+2CH_2+2H	1	806.4210	806.4229	2.4
y8	-1H_2O_-1CO_2H_2	1	793.3532	793.3555	2.8
y9	-2H_2O_-1CO_2H_2_+2H	1	864.3906	864.3926	2.3
y10	-2H_2O_-1CO_2H_2_+2H	1	963.4602	963.4610	0.8
y11	-2H_2O_-1CO_2H_2_+2H	1	1034.4963	1034.4981	1.7
y12	-3H_2O_-1CO_2H_2_+4H	1	1105.5319	1105.5352	3.0
y13	-3H_2O_-1CO_2H_2_+4H	1	1176.5683	1176.5723	3.4
y14	-4H_2O_-1CO_2H_2_+6H	1	1247.6045	1247.6094	4.0
y15	-4H_2O_-1CO_2H_2_+6H	1	1318.6418	1318.6465	3.6
y24	-8H_2O_-1CO_2H_2_+8H	2	1063.9910	1063.9932	2.1

Figure S26. Tandem MS/MS analysis for kebanetide A1 (**4**).
Source data are provided in MassIVE (DOI: 10.25345/C5PC2TM3Q).

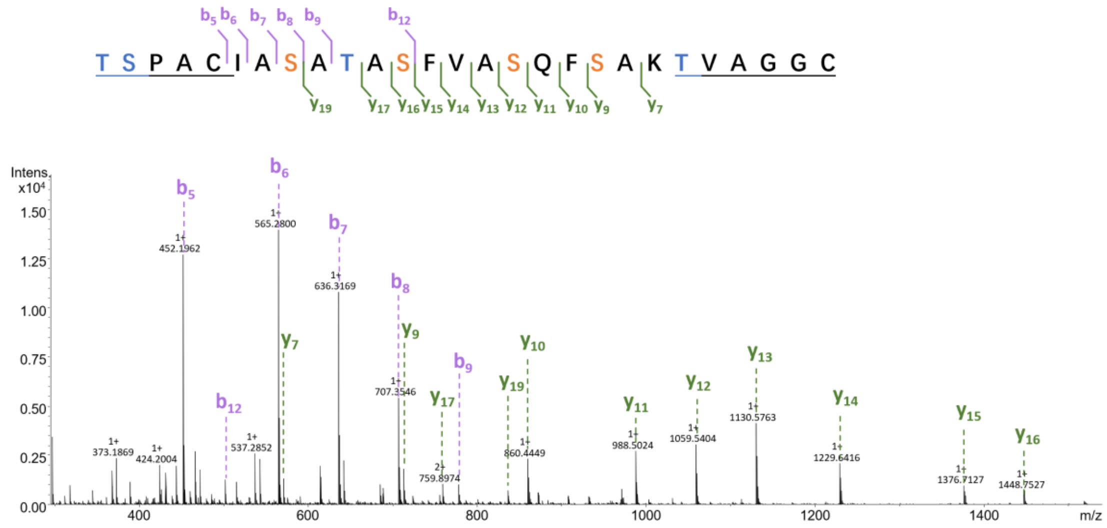

ions	modification	z	observed m/z	calculated m/z	Δ ppm
b5	-2H_2O_+2CH_2	1	452.1962	452.1962	0.0
b6	-2H_2O_+2CH_2	1	565.2800	565.2803	0.5
b7	-2H_2O_+2CH_2	1	636.3169	636.3174	0.8
b8	-3H_2O_+2CH_2_+2H	1	707.3546	707.3545	0.1
b9	-3H_2O_+2CH_2_+2H	1	778.3918	778.3916	0.2
b12	-5H_2O_+2CH_2_+4H	2	502.2540	502.2551	2.2
y7	-1H_2O_-1CO_2H_2	1	571.3001	571.3026	4.4
y9	-2H_2O_-1CO_2H_2_+2H	1	713.3760	713.3769	1.2
y10	-2H_2O_-1CO_2H_2_+2H	1	860.4449	860.4453	0.4
y11	-2H_2O_-1CO_2H_2_+2H	1	988.5024	988.5038	1.5
y12	-3H_2O_-1CO_2H_2_+4H	1	1059.5404	1059.5410	0.5
y13	-3H_2O_-1CO_2H_2_+4H	1	1130.5763	1130.5781	1.6
y14	-3H_2O_-1CO_2H_2_+4H	1	1229.6416	1229.6465	4.0
y15	-3H_2O_-1CO_2H_2_+4H	1	1376.7127	1376.7149	1.6
y16	-4H_2O_-1CO_2H_2_+6H	1	1447.7589	1447.7520	4.8
y17	-4H_2O_-1CO_2H_2_+6H	2	759.8974	759.8982	1.1
y19	-5H_2O_-1CO_2H_2_+6H	2	836.9372	836.9353	2.3

Figure S27. Tandem MS/MS analysis for kebanetide A2 (**5**).
Source data are provided in MassIVE (DOI: 10.25345/C5PC2TM3Q).

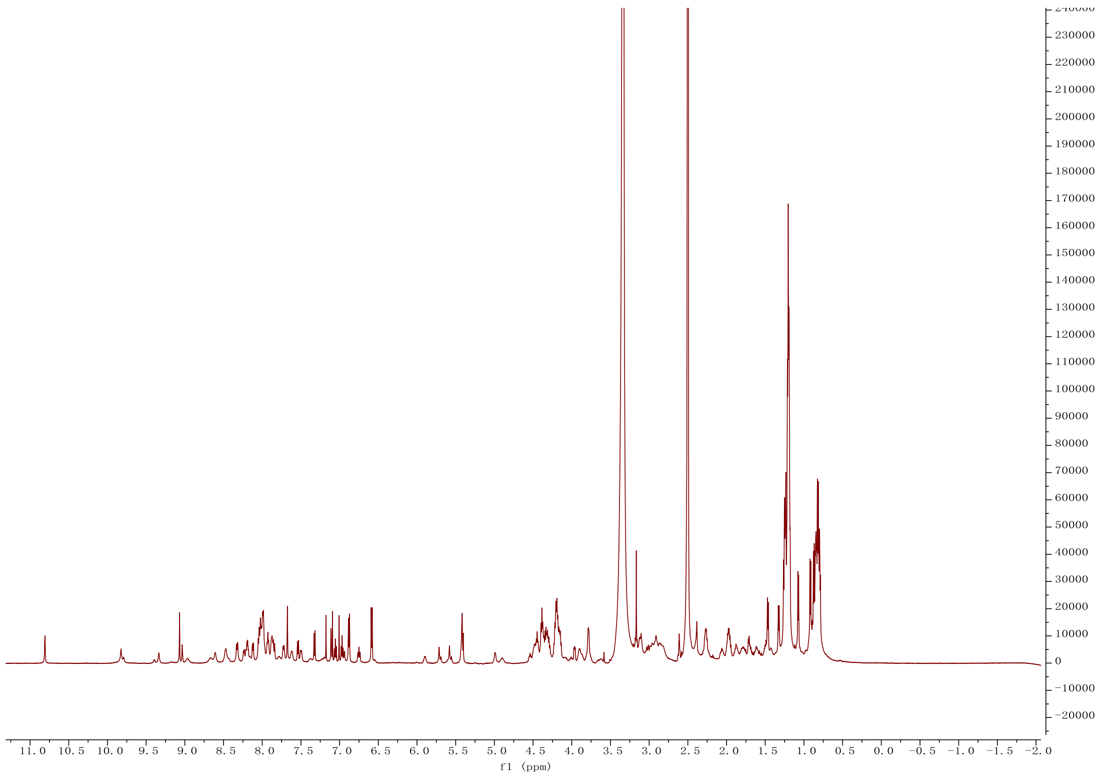

Figure S28. ^{1}H NMR spectrum (600 MHz, DMSO-*d6*) of kebanetideA1 (**4**).

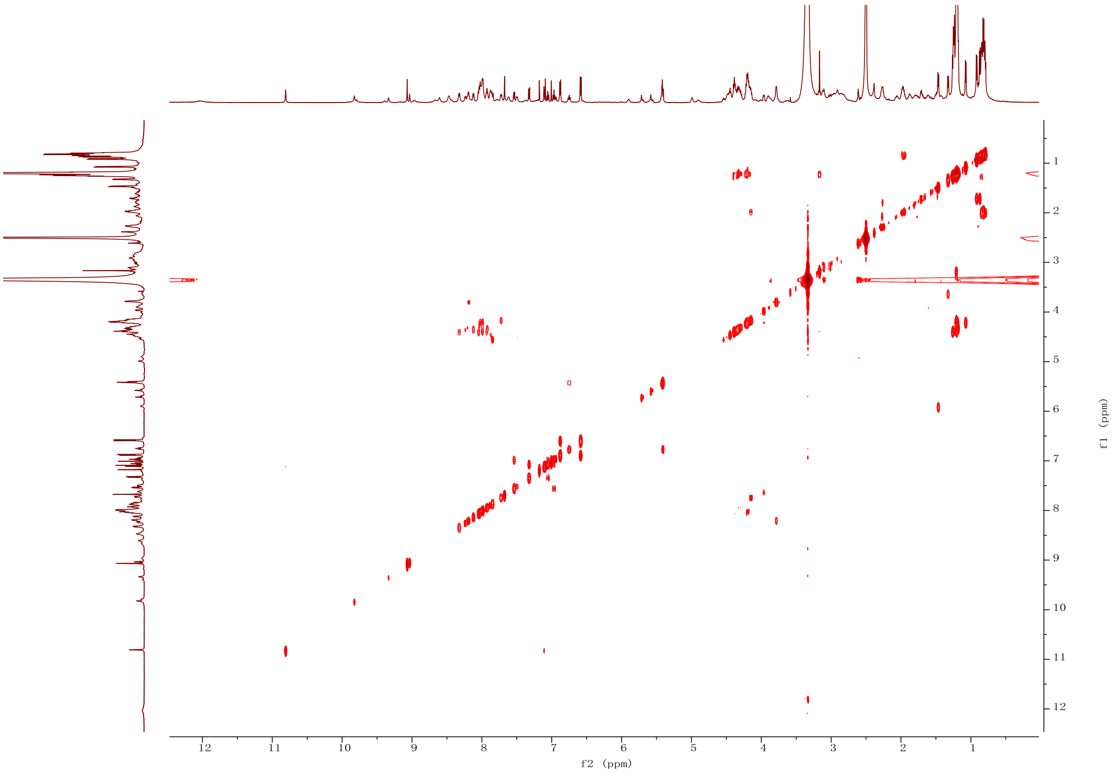

Figure S29. ^{1}H-^{1}H COSY spectrum (600 MHz, DMSO-*d6*) of kebanetide A1 (**4**).

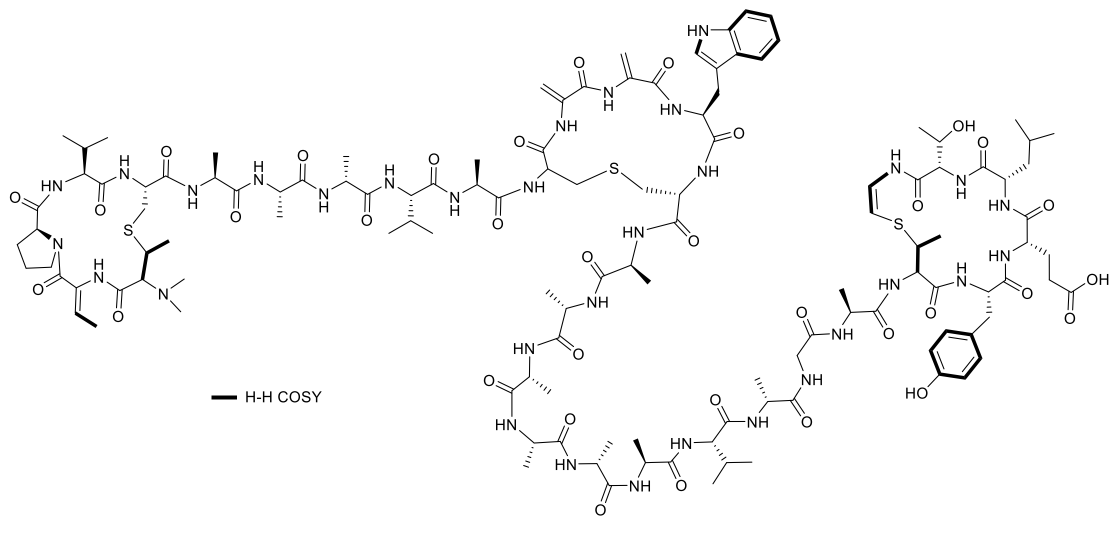

Figure S30. Key COSY correlations of kebanetide A1 (**4**).

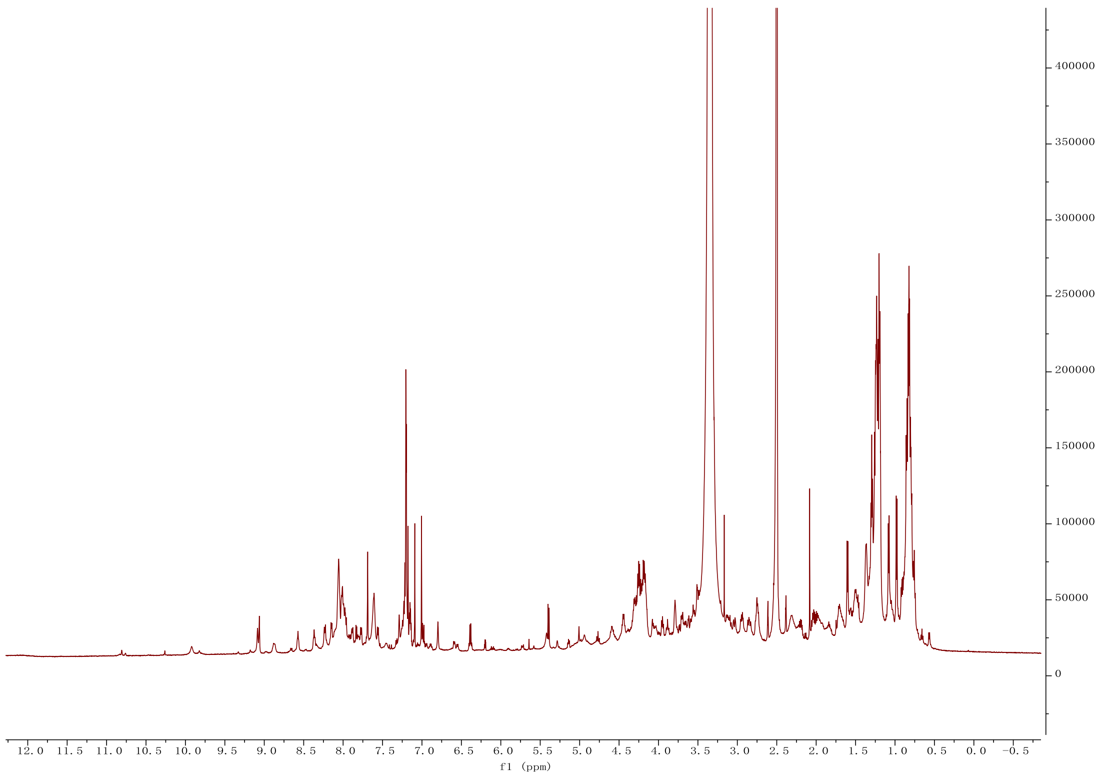

Figure S31. ^{1}H NMR spectrum (600 MHz, DMSO-*d6*) of kebanetide A2 (**5**).

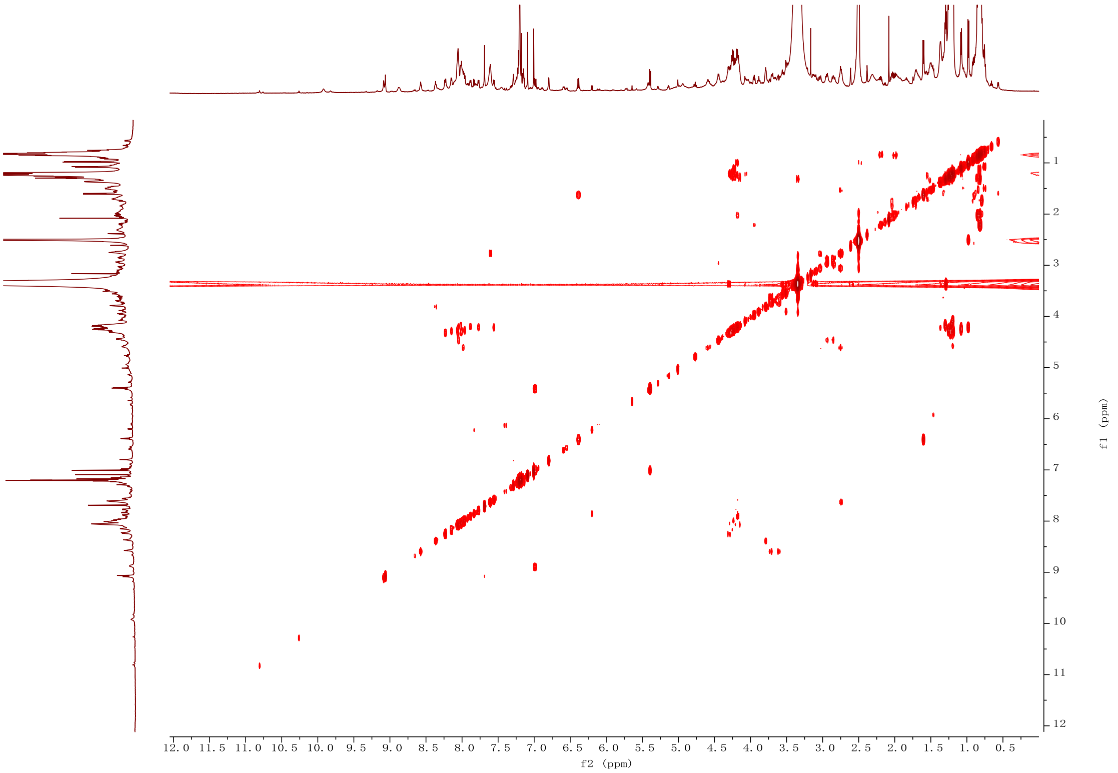

Figure S32. ^{1}H-^{1}H COSY spectrum (600 MHz, DMSO-*d6*) of kebanetide A2 (**5**).

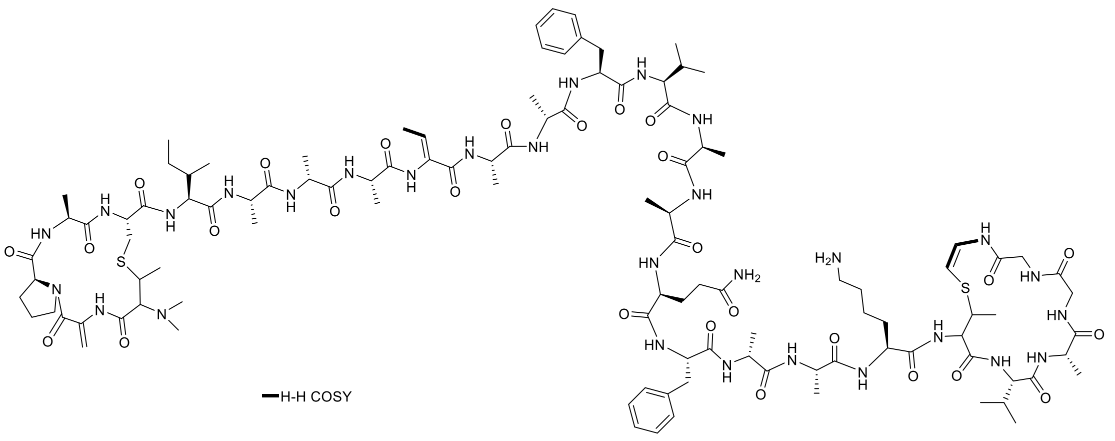

Figure S33. Key COSY correlations of kebanetide A2 (**5**).

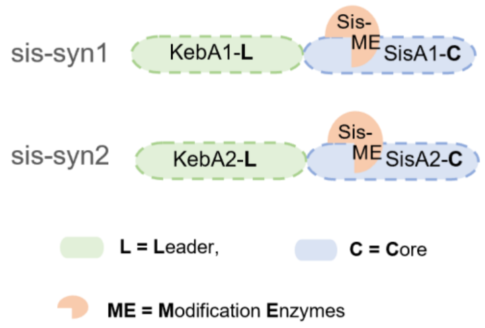

Figure S34. Chimeric precursor with maturase from *sis* BGC (constructs sis-syn1 and sis-syn2 design).
These two constructs both use *sis* family modification enzymes. The precursor Syn1 contains KebA1 leader and SisA1 core peptide. The precursor Syn2 contains KebA2 leader and SisA2 core peptide.

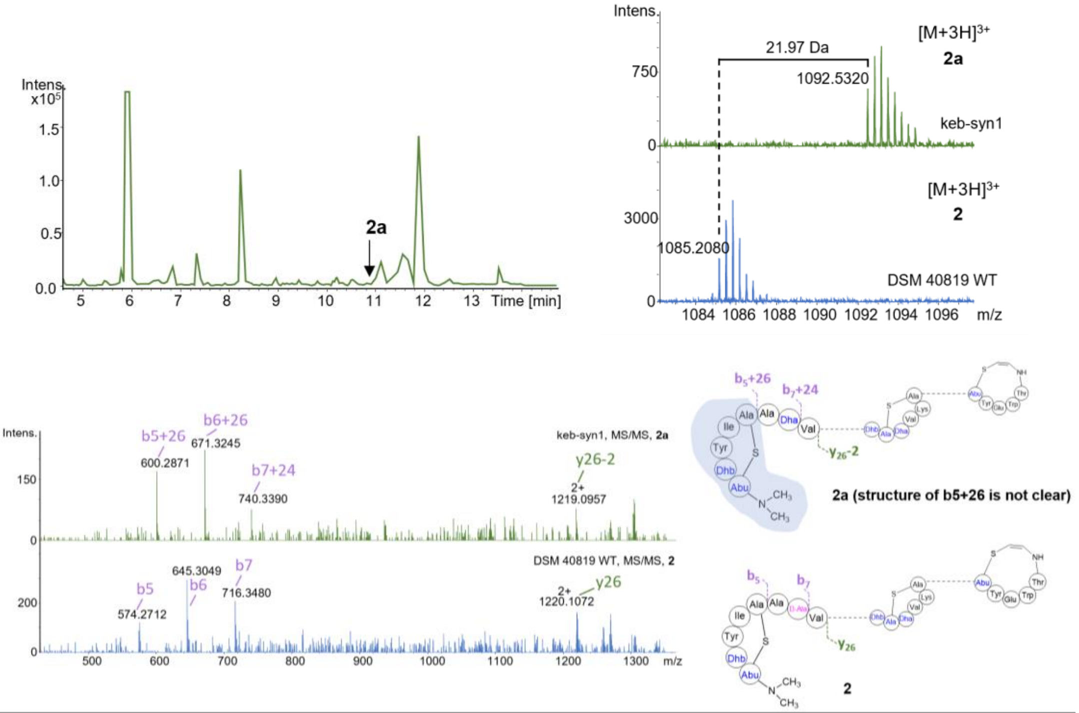

Figure S35. MS analysis for keb-syn1.

Compared to the wild type, keb-syn1 produce a compound **2** analog**, 2a**. We cannot predict the structure of **2a** based on the tandem mass. However, the fragment ions indicated that the b5 ion of compound **2a** is 26 Da heavier than that of compound **2**. The 7th residue of compound **2a** is Dha not D-Ala. y26 ion in **2a** is 2 Da lighter than that of in compound **2**, suggesting the presence of a Dha residue instead of D-Ala which cannot be precisely located due to the low intensity of the tandem mass. Source data are provided in MassIVE (DOI: 10.25345/C5PC2TM3Q).

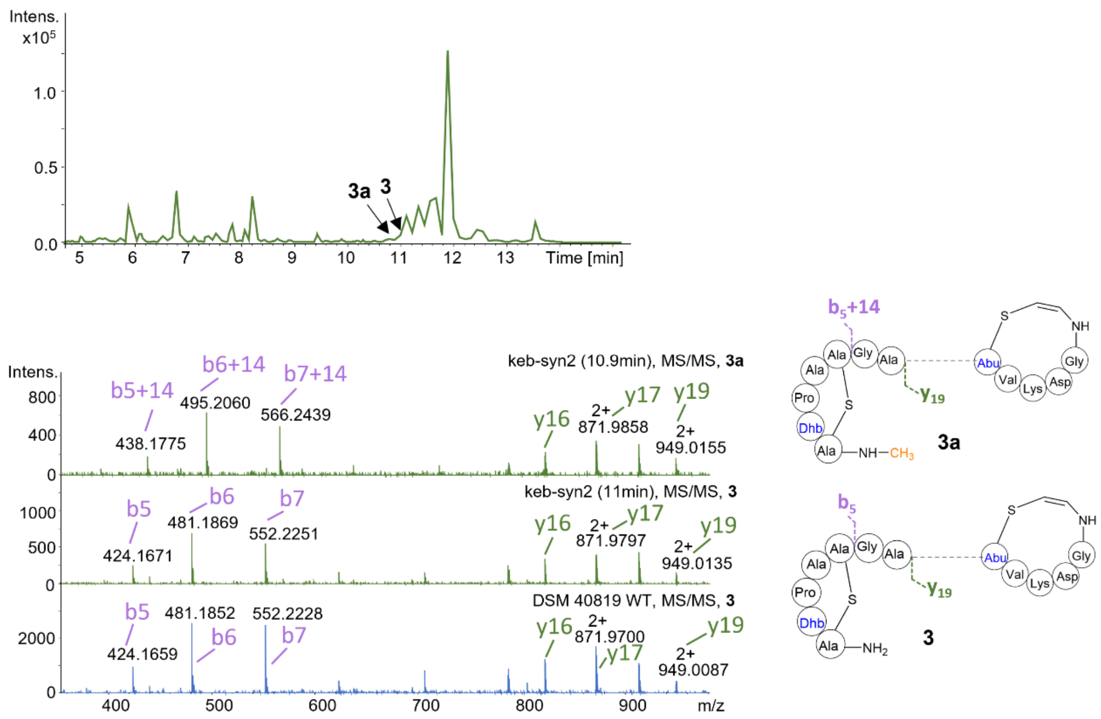

Figure S36. Retention time and tandem MS/MS analysis for keb-syn2.

Compared to the wild type, keb-syn2 produce two compounds, **3** and **3a**. The b5-b7 ions of compound **3a** are 14 Da heavier than that of compound **3**, indicating a methyl group at N-terminus. Source data are provided in MassIVE (DOI: 10.25345/C5PC2TM3Q).

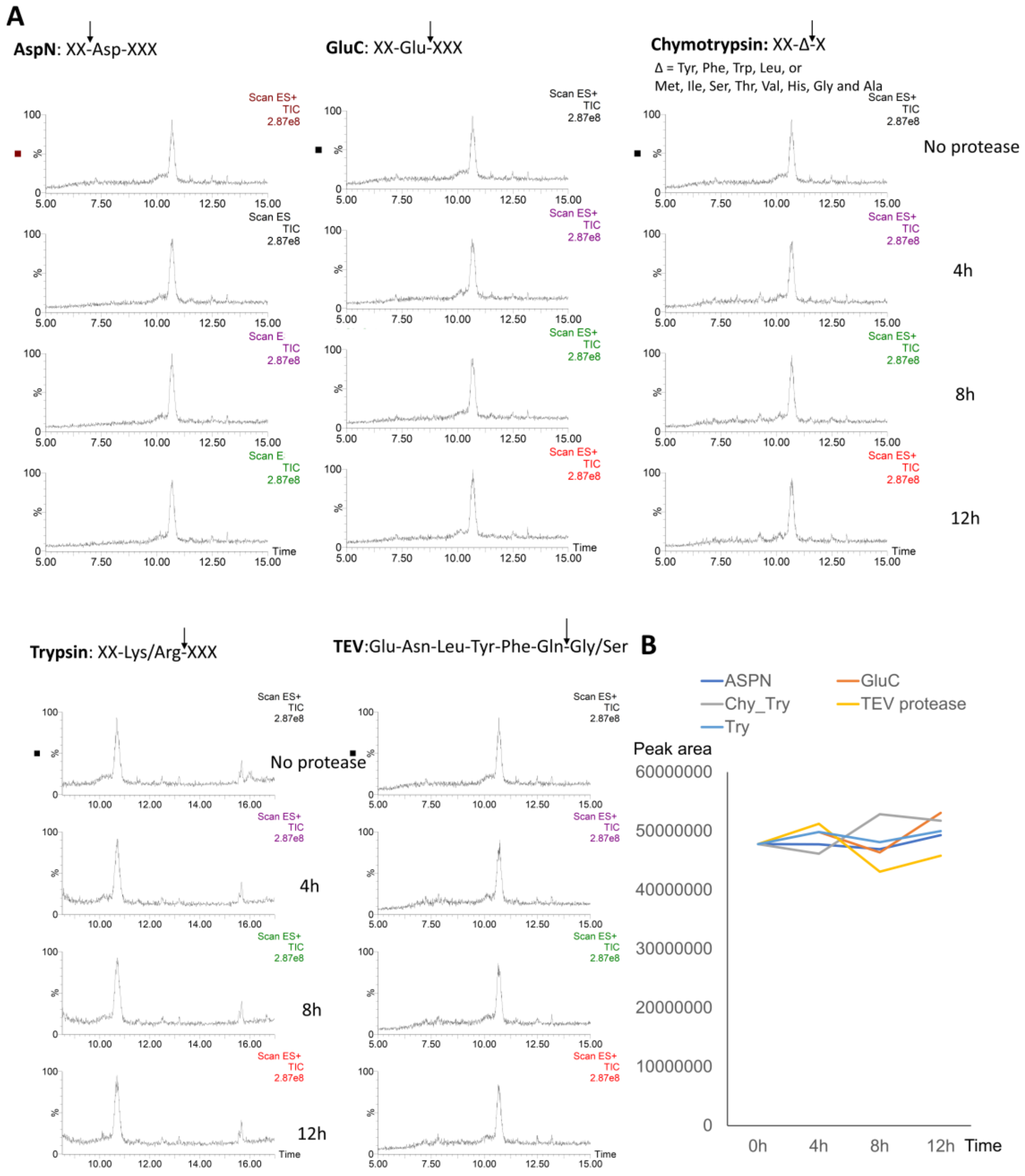

Figure S37. Proteolysis stability of massatide A (**1**).

A. Time-course analysis of protease-digested massatide A monitored by UPLC-LRMS. Black arrow denotes cleavage site of each protease. B. Time-to-peak area plot of each protease. No obvious peak area changes were observed in all the tested proteases, indicating massatide A exhibits proteolysis resistance against these proteases.

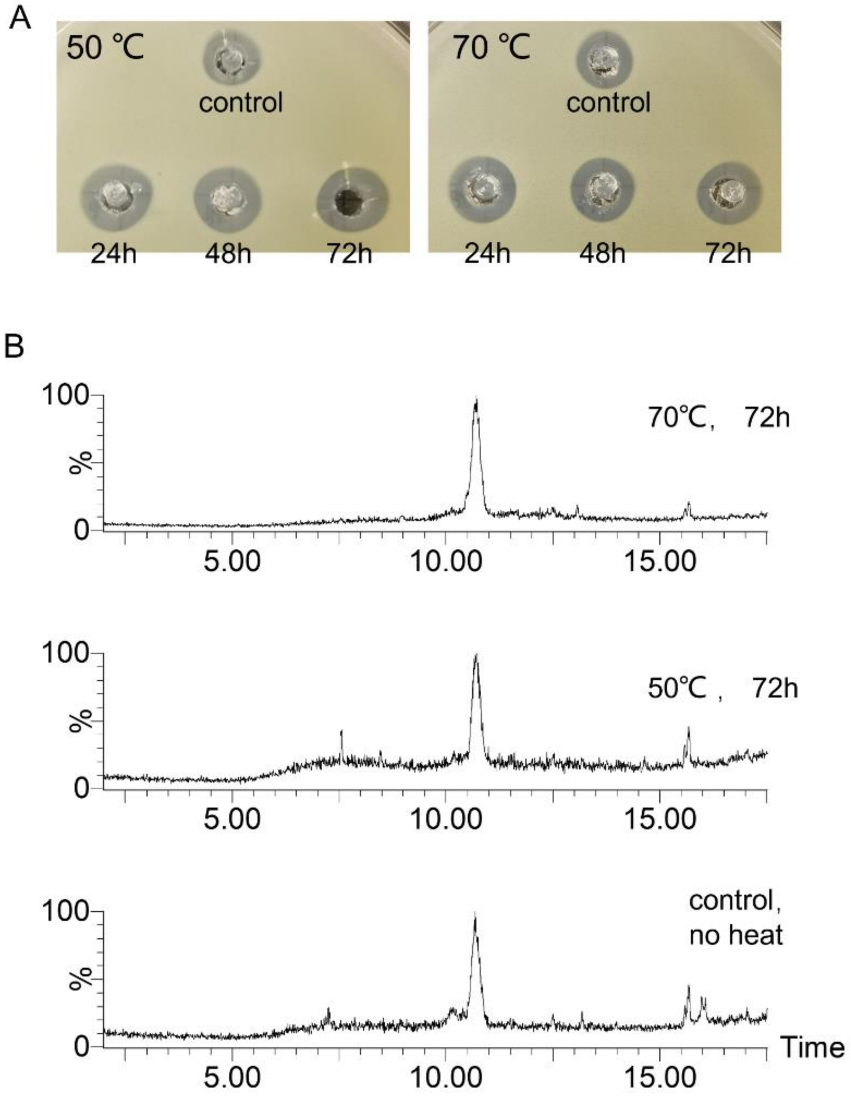

Figure S38. Thermal stability of massatide A (**1**).

A. Time-course activity analysis of massatide A. B. UPLC-LRMS analysis of massatide A after incubated at 50 ℃ and 70 ℃ for 72 h. No changes in retation time or *m/z* were observed after 72 h incubation under 70 ℃. Furthermore, no obvious changes in bioactivity were observed.

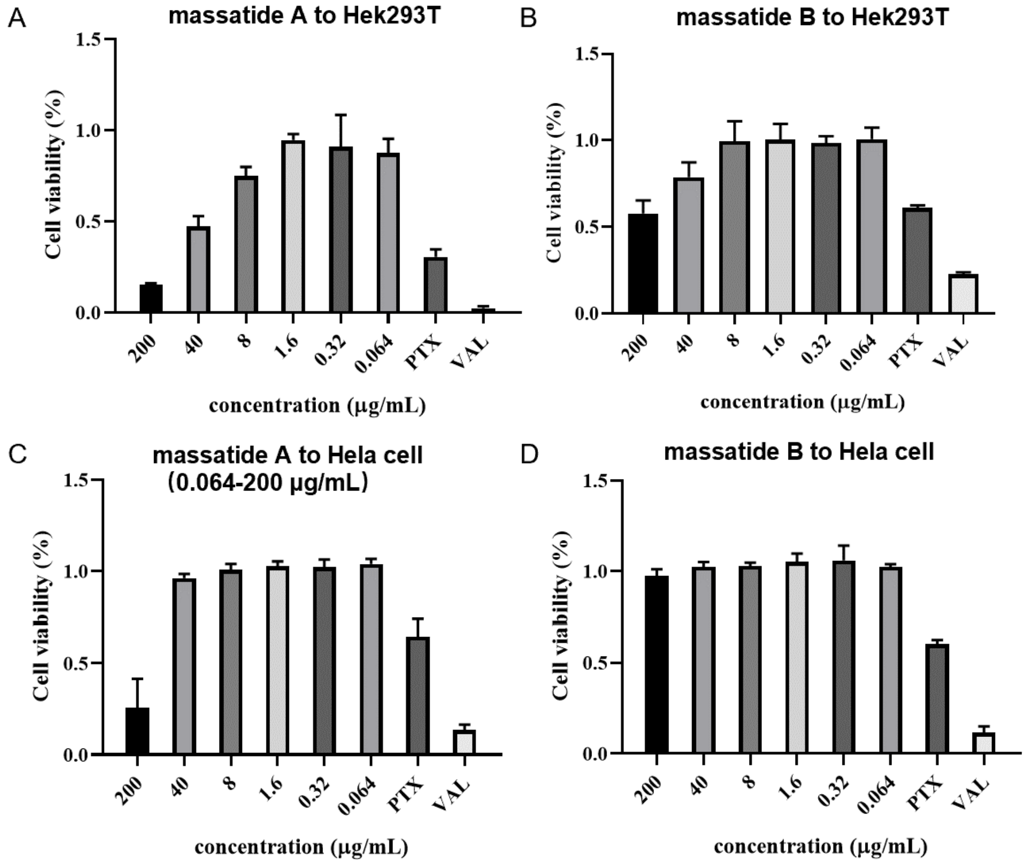

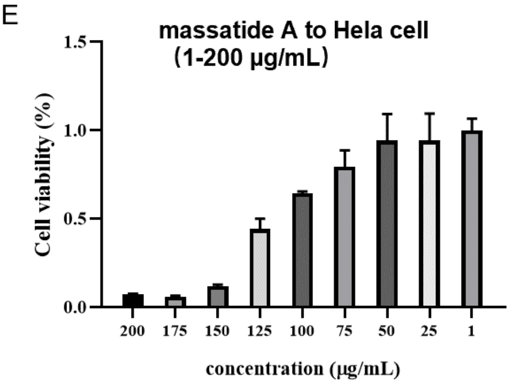

F

IC50 (μg/mL)

Cell lines	Hela	Hek293T
massatide A	136.3	25.9
massatide B	>200	85.5

Figure S39. Cytotoxicity of massatide A (**1**) and massatide B (**1a**).

A. Cytotoxicity of massatide A to Hek293T cell. B. Cytotoxicity of massatide B to Hek293T cell. C. Cytotoxicity of massatide A to Hela cell (test range 0.064-200μg/mL). Toxicity was only observed in 200 μg/mL group. D. Cytotoxicity of massatide B to Hela cell. E. Cytotoxicity of massatide A to Hela cell. (To calculate the IC50 value, test range was adjusted to 1-200μg/mL) F. IC50 value of massatides to Hela and Hek293T cell. Cell viability are graphed as mean + s.d. (n=3). Source data are provided as a Source Data file.

<table>
<tr><th>Compound</th><th>Structural features</th><th>Activity</th><th>Synthesis</th></tr>
<tr><td>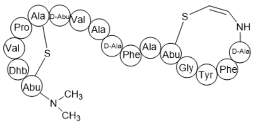
Massatide A</td><td>Two thioethers
AviCys motif
D-amino acid
N-di-methylation
MW = 1635 Da</td><td><table><tr><th></th><th>strains</th><th>MIC (ug/mL)</th></tr><tr><td>G+</td><td>S. aureus ATCC25923</td><td>0.5</td></tr><tr><td></td><td>B. subtilis 168</td><td>2</td></tr><tr><td></td><td>M. luteus DSM1790</td><td>0.06</td></tr><tr><td>G-</td><td>Escherichia coli DH5α</td><td>>128</td></tr><tr><td></td><td>Pseudomonas aeruginosa PAO1</td><td>>128</td></tr></table></td><td>biosynthesis</td></tr>
<tr><td>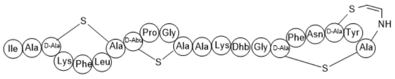
gallidermin</td><td>Four thioethers
AviCys motif
D-amino acid
Positively charged residues
MW = 2166 Da</td><td><table><tr><th></th><th>strains</th><th>MIC (ug/mL)</th></tr><tr><td>G+</td><td>S. aureus SG511</td><td>4</td></tr><tr><td></td><td>S. aureus E88</td><td>8</td></tr><tr><td></td><td>M. luteus ATCC9341</td><td>0.25</td></tr><tr><td></td><td>M. luteus 15957</td><td>0.5</td></tr><tr><td>G-</td><td>Escherichia coli ATCC11775</td><td>128</td></tr><tr><td></td><td>Pseudomonas aeruginosa BC19</td><td>128</td></tr></table></td><td>biosynthesis</td></tr>
<tr><td>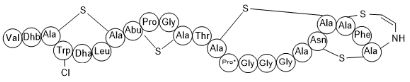
Pro* = 3,4-dihydroxy-proline, Microbisporicin A1
Pro* = 4-hydroxy-proline, Microbisporicin A2
Microbisporicin/NAI-107</td><td>Five thioethers
AviCys motif
D-amino acid
Halogenation
MW = A1, 2246 Da
A2, 2230 Da</td><td><table><tr><th></th><th>strains</th><th>MIC (ug/mL)</th></tr><tr><td>G+</td><td>S. aureus Atcc6538P</td><td><0.13</td></tr><tr><td></td><td>S. aureus VISA</td><td>2</td></tr><tr><td></td><td>E. faecalis</td><td>1</td></tr><tr><td>G-</td><td>Escherichia coli</td><td>>128</td></tr></table></td><td>biosynthesis</td></tr>
<tr><td>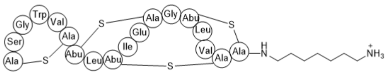
NVB302
(7-aminoheptylamido-deoxyactagardine B)</td><td>Four thioethers
D-amino acid
Halogenation
7-aminoheptylamido substitution
MW = 1971 Da</td><td><table><tr><th></th><th>strains</th><th>MIC (ug/mL)</th></tr><tr><td>G+</td><td>S. aureus MSSA</td><td>8-32</td></tr><tr><td></td><td>S. aureus MRSA</td><td>8-32</td></tr><tr><td></td><td>E. faecalis (van-sensitive)</td><td>4-8</td></tr><tr><td></td><td>E. faecalis VREF</td><td>2-16</td></tr><tr><td></td><td>S. epidermidis</td><td>2-32</td></tr></table></td><td>Chemical Semi-synthesis</td></tr>
</table>

Figure S40. The structural features, activity and synthesis of massatide A compared with other lanthipeptides in preclinical development.

References

1. Gust, B., Challis, G.L., Fowler, K., Kieser, T. & Chater, K.F. PCR-targeted Streptomyces gene replacement identifies a protein domain needed for biosynthesis of the sesquiterpene soil odor geosmin. *Proc. Natl. Acad. Sci. U.S.A.* **100**, 1541-1546 (2003).

2. Fujii, K., Ikai, Y., Oka, H., Suzuki, M. & Harada, K. A nonempirical method using LC/MS for determination of the absolute configuration of constituent amino acids in a peptide: Combination of Marfey's method with mass spectrometry and its practical application. *Anal. Chem.* **69**, 5146-5151 (1997).

3. Li, L. et al. The mildiomycin biosynthesis: initial steps for sequential generation of 5-hydroxymethylcytidine 5'-monophosphate and 5-hydroxymethylcytosine in Streptoverticillium rimofaciens ZJU5119. *Chembiochem* **9**, 1286-1294 (2008).

4. Wendt-Pienkowski, E. et al. Cloning, sequencing, analysis, and heterologous expression of the fredericamycin biosynthetic gene cluster from Streptomyces griseus. *J. Am. Chem. Soc.* **127**, 16442-16452 (2005).

5. Liang, H., Lopez, I.J., Sanchez-Hidalgo, M., Genilloud, O. & van der Donk, W.A. Mechanistic Studies on Dehydration in Class V Lanthipeptides. *ACS ACS Chem. Biol.* **17**, 2519-2527 (2022).
