# Supplementary material for: Rule-based omics mining reveals antimicrobial macrocyclic peptides against drug-resistant clinical isolates
Source: Nat Commun. 2024 Jun 8;15:4901. doi: 10.1038/s41467-024-49215-y (PMC11162475; doi:10.1038/s41467-024-49215-y)
Supplement: Supplementary file 4 — Supplementary Data 1-8 [file 41467_2024_49215_MOESM4_ESM.zip › Legends_of_Supplementary_data.docx]

Description of Additional Supplementary data Files：

Supplementary dataset 1:

The full list of genome information, flavoprotein sequences, putative precursor sequences, accession id of flavoproteins, strain names and strain kingdoms.

Supplementary dataset 2:

Sequence logos of 67 precursor clusters shown in the SSN. The file name format denotes ***cluster number***_***number of sequences***_***phyla***_***representative sequence***.

Supplementary dataset 3:

Phylogenetic analysis of 32 ACP BGC families.

Supplementary dataset 4:

The mass calculation table of *mat* BGC.

Supplementary dataset 5:

Tandem mass calculation table of *mat* BGC.

Supplementary dataset 6:

The mass calculation table of *sis* BGC (including tandem mass).

Supplementary dataset 7:

The mass calculation table of *keb* BGC (including tandem mass).

Supplementary dataset 8:

Protein sequence and accession ID of the BGC in this study.
