# Supplementary material for: Rule-based omics mining reveals antimicrobial macrocyclic peptides against drug-resistant clinical isolates
Source: Nat Commun. 2024 Jun 8;15:4901. doi: 10.1038/s41467-024-49215-y (PMC11162475; doi:10.1038/s41467-024-49215-y)
Supplement: Supplementary file 5 — Reporting Summary [file 41467_2024_49215_MOESM5_ESM.pdf]

nature portfolio

Corresponding author(s): Wenxuan Zhang, Xiaoyu Tang and Yong-Xin Li

Last updated by author(s): May 2, 2024

Reporting Summary

Nature Portfolio wishes to improve the reproducibility of the work that we publish. This form provides structure for consistency and transparency in reporting. For further information on Nature Portfolio policies, see our Editorial Policies and the Editorial Policy Checklist.

Statistics

For all statistical analyses, confirm that the following items are present in the figure legend, table legend, main text, or Methods section.

n/a	Confirmed	
☐	☒	The exact sample size (*n*) for each experimental group/condition, given as a discrete number and unit of measurement
☐	☒	A statement on whether measurements were taken from distinct samples or whether the same sample was measured repeatedly
☒	☐	The statistical test(s) used AND whether they are one- or two-sided<br>*Only common tests should be described solely by name; describe more complex techniques in the Methods section.*
☒	☐	A description of all covariates tested
☒	☐	A description of any assumptions or corrections, such as tests of normality and adjustment for multiple comparisons
☐	☒	A full description of the statistical parameters including central tendency (e.g. means) or other basic estimates (e.g. regression coefficient) AND variation (e.g. standard deviation) or associated estimates of uncertainty (e.g. confidence intervals)
☒	☐	For null hypothesis testing, the test statistic (e.g. *F*, *t*, *r*) with confidence intervals, effect sizes, degrees of freedom and *P* value noted<br>*Give P values as exact values whenever suitable.*
☒	☐	For Bayesian analysis, information on the choice of priors and Markov chain Monte Carlo settings
☒	☐	For hierarchical and complex designs, identification of the appropriate level for tests and full reporting of outcomes
☒	☐	Estimates of effect sizes (e.g. Cohen's *d*, Pearson's *r*), indicating how they were calculated

Our web collection on statistics for biologists contains articles on many of the points above.

Software and code

Policy information about availability of computer code

Data collection	UPLC-HRMS data was collected using reverse phase column (Waters ACQUITY UPLC BEH C18, 130Å, 1.7 µm column) on Thermo Scientific UltiMate 3000 UHPLC system coupled with Bruker impact Mass Spectrometer. 1H NMR, 13C NMR, HSQC, 1H-1H COSY, TOCSY, and NOESY spectra were acquired on a Bruker Avance 600 MHz spectrometer with Cryoprobe, using dimethyl sulfoxide--d6 as solvent. The MIC values of clinical isolates were determined by Sichuan Primed Shines Bio-tech Co., Ltd. (China). Growth kinetics data was collected on SpectraMax iD3 (MOLECULAR DEVICES).
Data analysis	Genome datasets were obtained from the NCBI database (Date: 2021-08-11). SPECO (https://github.com/yxllab-hku/ACyPs_code/tree/main/speco) was used to predict the BGC. BiG-SCAPE (version 1.1.5 (2022-11-14)) was used for BGC similarity network construction, and Cytoscape (version 3.9.0) for precursor similarity network visualization. MMseqs2 (version: 67949d702dbfc6e5d54fdd0f14a9ab6740f11c32) was used to cluster precursor sequences. Mass calculation was conducted using Python script (https://github.com/yxllab-hku/ACyPs_code/tree/main/ms-calculation). NMR data were analyzed using MestReNova (version: 12.0.2-20910). Chemical structures were drawn using ChemBioDraw (version 19.0). HRMS data was analyzed using Bruker DataAnalysis 4.3 (×64) .

For manuscripts utilizing custom algorithms or software that are central to the research but not yet described in published literature, software must be made available to editors and reviewers. We strongly encourage code deposition in a community repository (e.g. GitHub). See the Nature Portfolio guidelines for submitting code & software for further information.

Data

Policy information about availability of data

All manuscripts must include a data availability statement. This statement should provide the following information, where applicable:

- Accession codes, unique identifiers, or web links for publicly available datasets
- A description of any restrictions on data availability
- For clinical datasets or third party data, please ensure that the statement adheres to our policy

The BGCs identified in this study are available in Supplementary dataset 1-3. mass calculation in this study are available in supplementary dataset 4-7. Data of time-killing assay and growth kinetics are available in Source data file. Protein and nucleotide sequences of all the characterized BGCs in this study are available in Supplementary Table S3 and supplementary dataset 8. The MS data generated in this study have been deposited in MassIVE (DOI: 10.25345/C5PC2TM3Q, https://massive.ucsd.edu/ProteoSAFe/dataset.jsp?accession=MSV000094648). The raw data for time-killing assay, mouse model and cytotoxicity are provided in source data file.

Code Availability
Python scripts used for BGC identification and mass calculation can be found at (https://github.com/yxllab-hku/ACyPs_code). The code was also linked to Zenodo (https://doi.org/10.5281/zenodo.11099314).

Research involving human participants, their data, or biological material

Policy information about studies with human participants or human data. See also policy information about sex, gender (identity/presentation), and sexual orientation and race, ethnicity and racism.

Reporting on sex and gender	N/A
Reporting on race, ethnicity, or other socially relevant groupings	N/A
Population characteristics	N/A
Recruitment	N/A
Ethics oversight	N/A

Note that full information on the approval of the study protocol must also be provided in the manuscript.

Field-specific reporting

Please select the one below that is the best fit for your research. If you are not sure, read the appropriate sections before making your selection.

☒ Life sciences ☐ Behavioural & social sciences ☐ Ecological, evolutionary & environmental sciences

For a reference copy of the document with all sections, see nature.com/documents/nr-reporting-summary-flat.pdf

Life sciences study design

All studies must disclose on these points even when the disclosure is negative.

Sample size	For animal studies, sample size was chosen based on prior experience. For acute toxicity study, four mice in each group were used. For survival analysis in septicemia model, five mice in each group were used as difference in survival with treatment is dramatic.
Data exclusions	No data was excluded.
Replication	For standard microbiological assays, MICs and time-killing assay, experiments were performed in biological triplicate. In animal studies, multiple mice were included in each group.
Randomization	Mice were randomly divided into each group for infection and treatment. Other experiments in this study would not benefit from nor require randomization.
Blinding	Blinding was not relevant to this study, as the results were quantitative and not subjective.

Reporting for specific materials, systems and methods

We require information from authors about some types of materials, experimental systems and methods used in many studies. Here, indicate whether each material, system or method listed is relevant to your study. If you are not sure if a list item applies to your research, read the appropriate section before selecting a response.

Materials & experimental systems

n/a	Involved in the study
☒	☐ Antibodies
☐	☒ Eukaryotic cell lines
☒	☐ Palaeontology and archaeology
☐	☒ Animals and other organisms
☒	☐ Clinical data
☒	☐ Dual use research of concern
☒	☐ Plants

Methods

n/a	Involved in the study
☒	☐ ChIP-seq
☒	☐ Flow cytometry
☒	☐ MRI-based neuroimaging

Eukaryotic cell lines

Policy information about cell lines and Sex and Gender in Research

Cell line source(s)	All cell lines were obtained from ATCC: Hela (CCL-2), Hek293T (Human embryonic kidney, CRL3216).
Authentication	None of the cell lines were additionally authentication.
Mycoplasma contamination	Cell lines were not additionally tested for mycoplasma.
Commonly misidentified lines (See ICLAC register)	No commonly misidentified lines were used in this study.

Animals and other research organisms

Policy information about studies involving animals; ARRIVE guidelines recommended for reporting animal research, and Sex and Gender in Research

Laboratory animals	6-week-old specific pathogen-free male ICR mice weighing 18-20 g were used for all animal studies. Mice were housed at 22 degree celsius and relative humidity 50%, with a 12:12h light:dark cycle.
Wild animals	No wild animals were used in this study.
Reporting on sex	No sex- and gender-based analyses were conducted in this study. Male mice were exclusively utilized for two primary reasons:<br>(1) Considering the influence of hormone levels on animal behavior, we opted for male mice due to the relative stability of testosterone levels in the absence of fights or mating behaviors, compared to the fluctuating estrogen and progesterone levels in female mice over short periods.<br>(2) Given the limited amount of compound 1, male mice were selected to mitigate potential individual differences attributed to gender.
Field-collected samples	No field-collected samples were included in this study.
Ethics oversight	Animal studies were approved by the Animal Care and Use Committee of the Institute of Materia Medica, Chinese Academy of Medical Sciences, and Peking Union Medical College (Beijing, China)

Note that full information on the approval of the study protocol must also be provided in the manuscript.

Plants

Seed stocks	N/A
Novel plant genotypes	N/A
Authentication	N/A
